# The effector program of human CD8 T cells supports tissue remodeling

**DOI:** 10.1084/jem.20230488

**Published:** 2024-01-16

**Authors:** Michael Delacher, Lisa Schmidleithner, Malte Simon, Philipp Stüve, Lieke Sanderink, Agnes Hotz-Wagenblatt, Marina Wuttke, Kathrin Schambeck, Brigitte Ruhland, Veronika Hofmann, Sebastian Bittner, Uwe Ritter, Asmita Pant, Sara Salome Helbich, Morten Voss, Niels A. Lemmermann, Lisa Bessiri-Schake, Toszka Bohn, Andreas Eigenberger, Ayse Nur Menevse, Claudia Gebhard, Nicholas Strieder, Hinrich Abken, Michael Rehli, Jochen Huehn, Philipp Beckhove, Thomas Hehlgans, Henrik Junger, Edward K. Geissler, Lukas Prantl, Jens M. Werner, Christian Schmidl, Benedikt Brors, Charles D. Imbusch, Markus Feuerer

**Affiliations:** 1 Leibniz Institute for Immunotherapy, Regensburg, Germany; 2 Chair for Immunology, University Regensburg, Regensburg, Germany; 3 Chair for Interventional Immunology, University Regensburg, Regensburg, Germany; 4 Chair for Genetic Immunotherapy, University Regensburg, Regensburg, Germany; 5 Institute of Immunology, University Medical Center Mainz, Mainz, Germany; 6 Research Center for Immunotherapy, University Medical Center Mainz, Mainz, Germany; 7 Institute of Virology, University Medical Center Mainz, Mainz, Germany; 8 Faculty of Biosciences, Heidelberg University, Heidelberg, Germany; 9 Faculty of Medicine Heidelberg, Heidelberg University, Heidelberg, Germany; 10 Division of Applied Bioinformatics, German Cancer Research Center, Heidelberg, Germany; 11 Core Facility Omics IT and Data Management, German Cancer Research Center, Heidelberg, Germany; 12 Institute of Virology, University of Bonn, Bonn, Germany; 13 Department of Plastic, Hand- and Reconstructive Surgery, University Hospital Regensburg, Regensburg, Germany; 14 Department of Internal Medicine III, University Hospital Regensburg, Regensburg, Germany; 15 Department of Experimental Immunology, Helmholtz Centre for Infection Research, Braunschweig, Germany; 16 Hannover Medical School, Hannover, Germany; 17 RESIST, Cluster of Excellence 2155, Hannover Medical School, Hannover, Germany; 18 Department of Surgery, University Hospital Regensburg, Regensburg, Germany; 19 National Center for Tumor Diseases, Heidelberg, Germany; 20 German Cancer Consortium, German Cancer Research Center, Heidelberg, Germany

## Abstract

CD8 T lymphocytes are classically viewed as cytotoxic T cells. Whether human CD8 T cells can, in parallel, induce a tissue regeneration program is poorly understood. Here, antigen-specific assay systems revealed that human CD8 T cells not only mediated cytotoxicity but also promoted tissue remodeling. Activated CD8 T cells could produce the epidermal growth factor receptor (EGFR)-ligand amphiregulin (AREG) and sensitize epithelial cells for enhanced regeneration potential. Blocking the EGFR or the effector cytokines IFN-γ and TNF could inhibit tissue remodeling. This regenerative program enhanced tumor spheroid and stem cell–mediated organoid growth. Using single-cell gene expression analysis, we identified an AREG^+^, tissue-resident CD8 T cell population in skin and adipose tissue from patients undergoing abdominal wall or abdominoplasty surgery. These tissue-resident CD8 T cells showed a strong TCR clonal relation to blood PD1^+^TIGIT^+^ CD8 T cells with tissue remodeling abilities. These findings may help to understand the complex CD8 biology in tumors and could become relevant for the design of therapeutic T cell products.

## Teaser

This work reveals that human effector CD8 T cells not only mediate cytotoxicity but also promote tissue remodeling. The remodeling potential was demonstrated in different systems, including a primary organoid model and antigen-specific assays.

## Introduction

Tissue regeneration and remodeling are critical functions to respond to tissue damage caused by infection, inflammation, trauma, or other insults, but excessive repair can also lead to fibrosis and support tumor growth (Duffield et al., 2013; Foster et al., 2018). Regeneration and remodeling can be promoted by soluble mediators, which include ligands of the epidermal growth factor (EGF) receptor such as amphiregulin (AREG) and transforming growth factor α (TGFα; Freed et al., 2017; Janes et al., 2006; Schneider et al., 2008; Zaiss et al., 2015). On a cellular level, different lineages can promote tissue regeneration, including stem cells, fibroblasts, differentiated parenchymal cells such as epithelial cells, and innate immune cells including tissue-resident macrophages (Dekoninck and Blanpain, 2019; Wynn and Vannella, 2016). In addition, lymphocytes have been shown to induce or support tissue repair. In this context, CD4^+^ regulatory T (Treg) cells with tissue-regenerative abilities have been described in different murine tissues under homeostasis and in diseases (Braband et al., 2022; Panduro et al., 2016). Their differentiation and progenitor development was first explored in mouse (Campbell and Rudensky, 2020; Delacher et al., 2017, 2020; Li et al., 2018), and recently, human tissue Treg cells with a tissue-repair program have been described as an effector-like Treg cell subpopulation (Delacher et al., 2021).

CD8 T cells are commonly viewed as cytotoxic T cells with important functions in the defense against infections and cancer. Therefore, cell products containing CD8 T cells are an approved therapy against certain types of cancers, e.g., when modified with chimeric-antigen-receptors (CARs; Larson and Maus, 2021). In cancer and under chronic inflammatory conditions, CD8 T cells can differentiate into a dysfunctional state called exhaustion (Philip and Schietinger, 2021; van der Leun et al., 2020). This state is characterized by the expression of several markers including PD1, TOX, and CD39, and a progressive reprogramming of the chromatin landscape, which leads to impaired effector molecule expression, proliferation, and survival (Alfei et al., 2019; Kallies et al., 2020; Khan et al., 2019; Philip et al., 2017; Scott et al., 2019). However, PD1 and TOX expression are not exclusive to the state of exhaustion as their expression is also associated with CD8 T cell activation (McLane et al., 2019; Sekine et al., 2020). Recent reports suggest the presence of an auto-aggressive CD8 T cell type in non-alcoholic steatohepatitis (NASH) and liver hepatocellular carcinoma (HCC, liver cancer), which displays markers for exhaustion, tissue residency, and effector function. Rather than mediating an anticancer effector function, this CD8 cell type has been described to promote fibrosis and tumor progression in a NASH mouse model by secreting TNF (Dudek et al., 2021; Pfister et al., 2021).

In this study, we examined whether human CD8 T cells can induce a tissue regeneration program. Our findings indicate that the human CD8 effector program not only leads to tumor cell killing but also supports tissue remodeling abilities, e.g., by promoting stem cell–mediated organoid growth. Our data suggest that IFN-γ, TNF, and AREG released by human CD8 T cells contribute to this underestimated effector arm of human CD8 T cells.

## Results

### Support of tissue regeneration and target cell killing by human CD8 T cells

**Figure 1.**
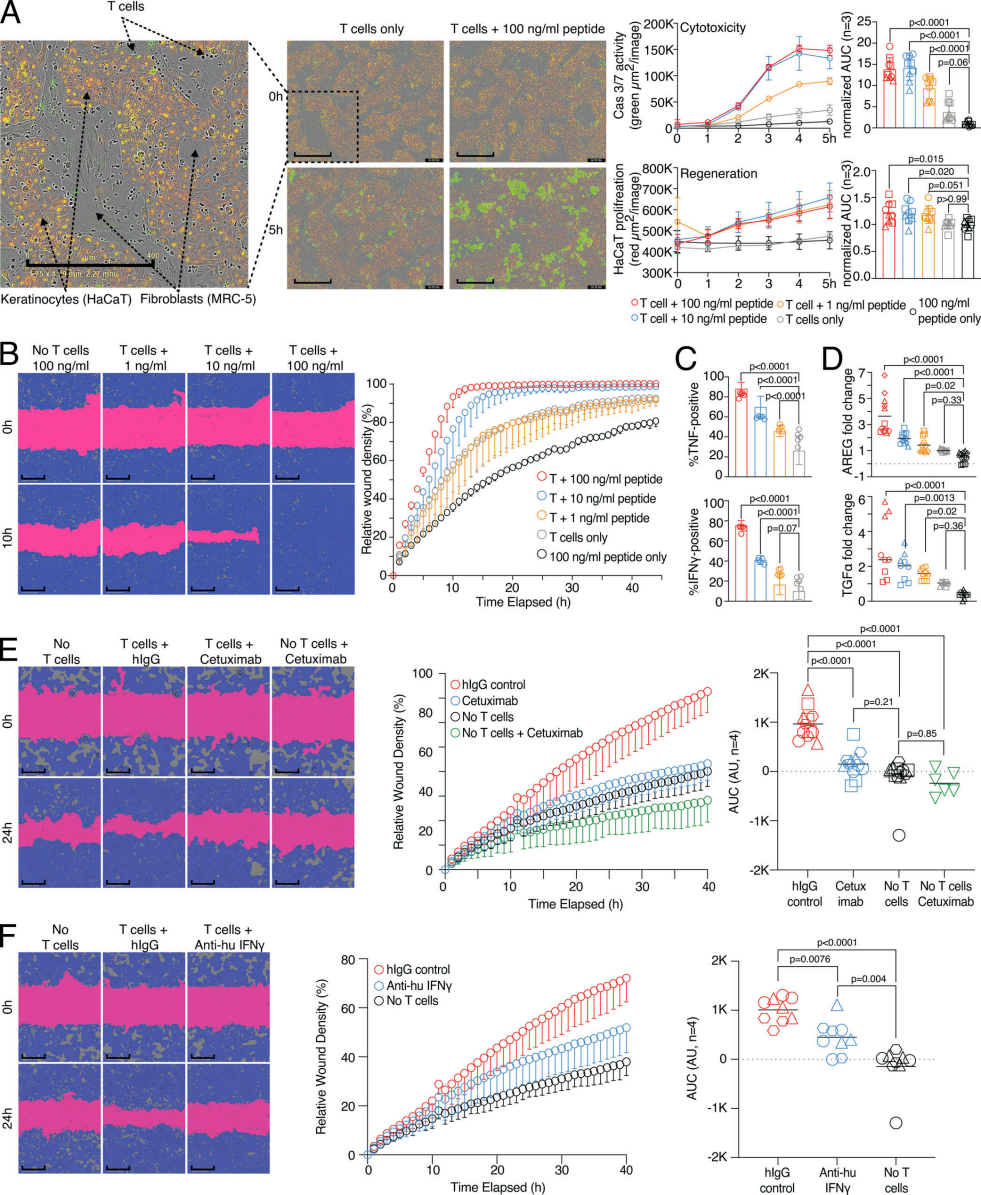
**Wound healing and target killing are both effector mechanisms of CD8 T cells. (A)** Combined proliferation and killing assay in the presence of influenza-specific CD8 T cells and varying amounts of pulsed peptides on MRC-5 and HaCaT cells seeded in a 30:70 ratio. Left, representative image with labels (0 versus 5 h; T cells only versus T cells + 100 ng/ml peptide). Right, representative quantification of Cas3/7 activity (green) and HaCaT proliferation (red) with titrated amounts of influenza peptide, with statistical verification across experiments using normalized area under the curve (AUC, *n* = 3, one-way ANOVA, symbols indicate individual experiments). Scale bars = 400 µm; enhanced for improved visibility. **(B)** SN from A tested in a wound healing assay with HaCaT cells; representative example with additional experiments in Fig. S1 (*n* = 3). Scale bars = 400 µm; enhanced for improved visibility. Reuse of the B panel in schematic of Fig. S1 A. **(C)** Measurement of intracellular TNF and IFN-γ in influenza-specific T cells used in the combined proliferation and killing assay, representative stainings (*n* = 12, one-way ANOVA), gating in Fig. S1. **(D)** Fold induction of AREG and TGFα in SNs from (A) (*n* = 3–4, one-way ANOVA, symbols indicate individual experiments) Individual experiments in Fig. S1. **(E and F)** Effect of using Cetuximab or anti-IFN-γ on wound healing capacity of SNs from (A) using background-corrected AUCs (*n* = 4, one-way ANOVA of AUC, symbols indicate individual experiments). Scale bars = 400 µm; enhanced for improved visibility. All data derived from three or more independent experiments.

**Figure S1.**
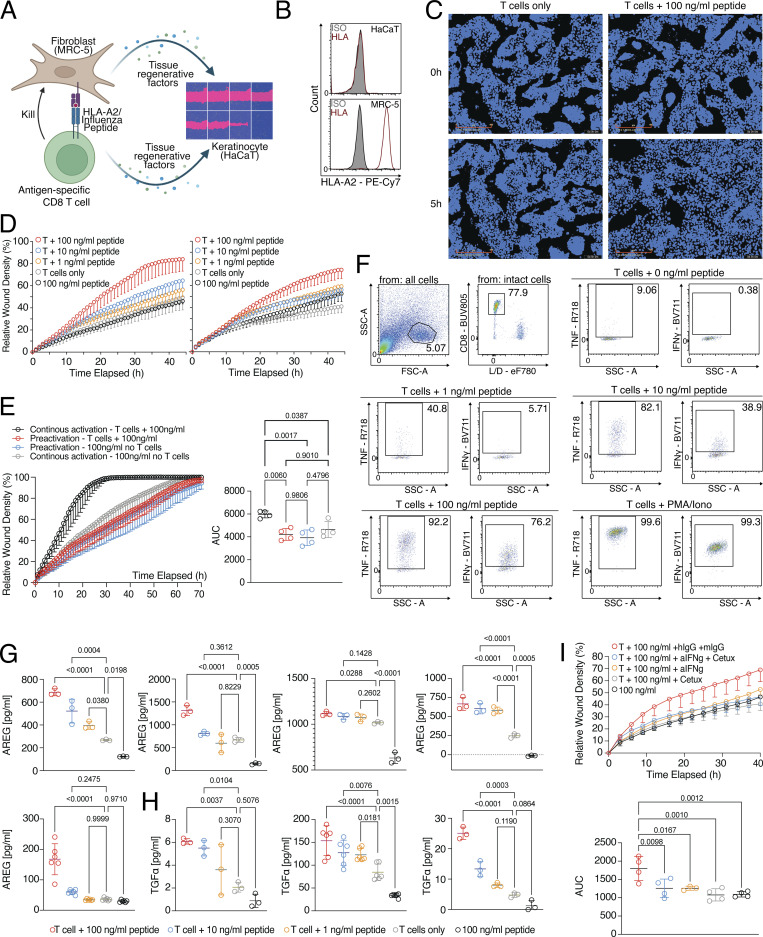
**Wound healing scratch assay. (A)** Schematic of three-cell-type system with HLA-A2^+^ fibroblast cells (MRC-5) presenting influenza peptide to influenza-specific CD8 T cells, and HLA-A2^−^ epithelial cells (HaCaT cells, keratinocytes). Visualization was created with BioRender using a graphical element from Fig. 1 B. **(B)** Expression of HLA-A2 in HaCaT versus MRC-5 cell lines. **(C)** Depiction of proliferation mask of HaCaT cells in a coculture with MRC-5 cells seeded in a 30:70 HaCaT:MRC-5 cell ratio pulsed with 0 (left) or 100 ng/ml (right) influenza peptide and cocultured with influenza-specific T cells after 0 (top) or 5 (bottom) h. Raw images are depicted in Fig. 1 A. Scale bars = 400 µm; enhanced for improved visibility. **(D)** MRC-5 and HaCaT cells were seeded in a 30:70 ratio and pulsed with varying amounts of influenza peptide for 1 h. Cells were cultured in the presence of influenza-specific T cells and cell-free SN was tested in a wound healing assay with HaCaT cells; two independent assay results (*n* = 3). **(E)** MRC-5 and HaCaT cells were seeded in a 30:70 ratio and pulsed with 100 ng/ml influenza peptide for 1 h. Cells were cultured in the presence or absence of influenza-specific T cells o/n. Cells were then either washed 3× and cultured o/n with fresh medium in the absence of influenza-specific T cells (preactivation) or left untouched (continuous activation). After an additional 24 h incubation period, cell-free SN was harvested and tested in a wound healing assay with HaCaT cells (*n* = 4). **(F)** Measurement of intracellular TNF and IFN-γ in influenza-specific T cells used in the combined proliferation and killing assay; representative gating. **(G and H)** Individual, unnormalized experiments of AREG (G) or TGFα (H) ELISA from Fig. 1 D. **(I)** MRC-5 and HaCaT cells were seeded in a 30:70 ratio and pulsed with 0 or 100 ng/ml influenza peptide for 1 h. Cells were cultured in the presence of influenza-specific T cells and cell-free SN was tested in a wound healing assay with HaCaT cells in the presence of aIFNγ, Cetuximab, aIFNγ, and Cetuximab or IgG control (*n* = 4).

To functionally understand human CD8 T cells with respect to promoting a regenerative potential versus cytotoxic effector functions, we established antigen-specific assay systems. We introduced a three-cell-type system where HLA-A2^+^ fibroblast cells (MRC-5) were able to present influenza virus peptide to donor-derived influenza-specific CD8 T cells in the presence of HLA-A2^−^ epithelial cells (keratinocytes, HaCaT cells), simulating an in vitro tissue environment with self-organized fibroblast and epithelial cell structures (Fig. 1 A and Fig. S1, A–C). In this assay, influenza peptide–presenting fibroblasts were killed by activated CD8 T cells in a dose-dependent manner, reflecting a readout for cytotoxicity (Fig. 1 A, upper right panel). However, in parallel, the number of fluorescence-labeled epithelial cells increased, also dose dependently, serving as a readout for tissue regeneration potential (Fig. 1 A, lower right panel). To exclude that epithelial cell growth increased merely based on the loss of influenza peptide–presenting fibroblasts in our three-cell-type system, cell-free supernatant (SN) was collected. This SN was then used in an in vitro live cell imaging wound healing assay with epithelial cells, which showed an influenza peptide dose-dependent increase in wound healing capacity (Fig. 1 B and Fig. S1 D). To determine whether a continuous presence of activated CD8 T cells is required for increased wound healing, cells of the three-cell-type system were pulsed with influenza peptide, as in Fig. 1 A. After 24 h of cocultivation, we then either removed the influenza-specific CD8 cells from the culture and washed the remaining cells and, thereby, removed the factors produced within the first 24 h (“preactivation”) or left the cells untouched (“continuous activation”). After an additional overnight (o/n) incubation, we harvested the SNs of both conditions and measured the wound healing potential with epithelial cells as in Fig. 1 B. No increase in wound healing potential was observed with SN generated from the preactivation condition, indicating that factors produced by the activated CD8 T cells were required for the wound healing potential (Fig. S1 E).

Increasing peptide concentrations induced a gradual activation of CD8 T cells as measured by TNF and IFN-γ production in this system (Fig. 1 C and Fig. S1 F). In addition, the three-cell-type system showed an influenza peptide dose-dependent production of TGFα and AREG (Fig. 1 D and Fig. S1, G and H). As both molecules, TGFα and AREG, are ligands of the EGF receptor (EGFR; Freed et al., 2017), we interfered with EGFR signaling by blocking the EGFR with a monoclonal blocking antibody (Cetuximab). Using Cetuximab in the culture abrogated the tissue-regenerative function completely (Fig. 1 E). To address whether there was an interdependency between effector and regeneration molecules, we blocked IFN-γ, which also impaired the wound healing capacity of the SN (Fig. 1 F). Blocking of EGFR and IFN-γ in combination did not further decrease wound healing in comparison to EGFR blocking by Cetuximab only (Fig. S1 I).

Together, these data indicate that antigen-specific activation of effector CD8 T cells in a tissue-like environment with fibroblast and epithelial cells can lead to target cell killing but also to the support of wound healing via the release of effector cytokines and EGFR ligands.

### Strong bystander activation of fibroblasts and epithelial cells by activated CD8 T cells

**Figure 2.**
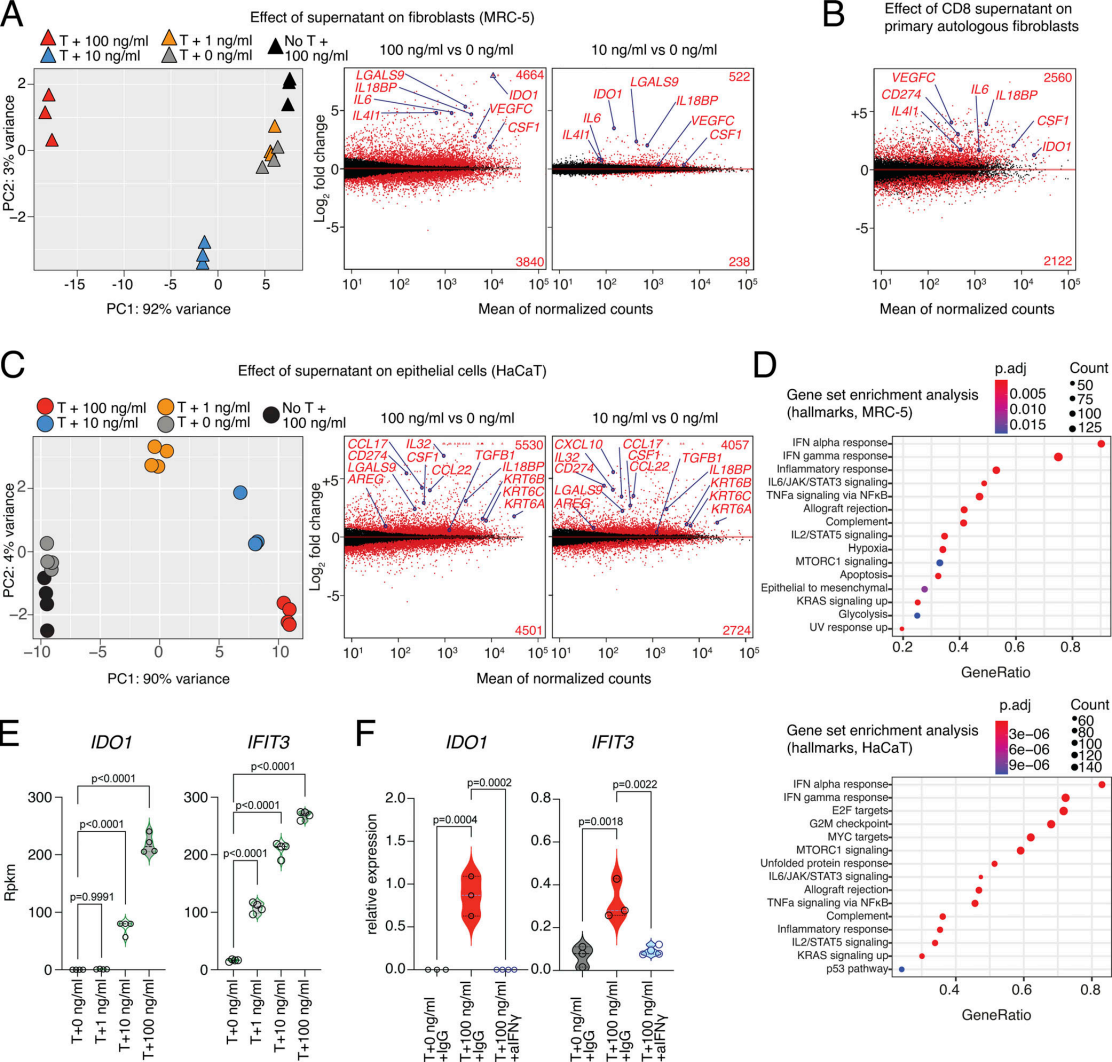
**CD8 T cells instruct other cell types to produce regenerative molecules. (A)** Left, PCA of RNA-seq of fibroblast (MRC-5) cells stimulated o/n with SN of MRC-5 cells pulsed with varying concentrations of influenza peptide and cultured with influenza-specific T cells o/n (*n* = 3); Right, MA-plots with number of DEGs (P_adj_ < 0.05) based on Deseq2 in red, several genes labeled. Gene expression data and statistical analysis in Table S1. **(B)** MA-plot of primary human fat fibroblasts stimulated with SN generated from autologous CD8 T cells isolated from fat tissue and stimulated o/n with IL-2 and beads or empty medium ctrl (*n* = 4). DEGs in red. Gene expression data and statistical analysis in Table S2. **(C)** Left, PCA of RNA-seq of epithelial cells (HaCaT) stimulated o/n with SN of MRC-5 cells pulsed with varying concentrations of influenza peptide and cultured with influenza-specific T cells o/n; Right, MA-plots with number of DEGs (Padj < 0.05) based on Deseq2 in red (*n* = 4), several genes labeled. Gene expression data and statistical analysis are in Table S3. **(D)** GSEA of MRC-5 fibroblasts (top) or HaCaT epithelial cells (bottom) stimulated o/n with SN of MRC-5 cells pulsed with 100 ng/ml of influenza peptide and cultured with influenza-specific T cells o/n. **(E)** Gene expression of *IDO1* and *IFIT3* in RNA-seq of epithelial cells (HaCaT) stimulated o/n with SN of MRC-5 cells pulsed with varying concentrations of influenza peptide and cultured with influenza-specific T cells o/n. Statistical analysis via Deseq2 (*n* = 3–4). **(F)** Relative expression of *IDO1* and *IFIT3* determined by quantitative PCR in epithelial cells (HaCaT) stimulated o/n in the presence of anti-IFN-γ or IgG control with SN of MRC-5 cells pulsed with 0 or 100 ng/ml of influenza peptide and cultured with influenza-specific T cells o/n. Statistical analysis via one-way ANOVA (*n* = 3–4).

Since we observed that factors produced by activated CD8 T cells were required for the wound healing potential, we were interested in understanding how these factors affected fibroblasts and epithelial cells on a molecular level. Therefore, we performed gene expression profiling of fibroblasts (MRC-5) stimulated with cell-free SN. This SN was produced by influenza-specific CD8 T cells cultured on fibroblasts that had been pulsed with different concentrations of influenza peptide (Fig. 2 A and Table S1). The comparison of the 100 ng/ml versus 0 ng/ml peptide-pulsed groups showed very strong activation of fibroblast cells by the cell-free SN of activated CD8 T cells, with 8,504 differentially expressed genes (DEGs). These differences included the induction of secondary effector molecules with the potential to interfere with a variety of cell types, such as *IL6* (proliferation of epithelial cells [Jeffery et al., 2017]), *VEGFC* (activation of endothelial cells [Rauniyar et al., 2018]), CSF1 (activation of tissue macrophages [Jones and Ricardo, 2013]), *IDO1*, and *IL4L1* (important enzymes for the production of aryl hydrocarbon receptor-ligands that could lead to epithelial cell regeneration [Metidji et al., 2018] and also important enzymes for the depletion of tryptophan, leading to suppression of T cell activation [Munn and Mellor, 2016]), and *IL18BP* and *LGALS9* (checkpoints of T cell and natural killer activation [Yang et al., 2021; Zhou et al., 2020]), which again increased in a dose-dependent manner (Fig. 2 A). To confirm that such a bystander activation effect of factors produced by primary human CD8 T cells isolated directly from healthy tissues can occur also on autologous primary human fibroblasts from the same tissue, CD8 T cells and fibroblasts were sorted from subcutaneous fat tissue of five donors. CD8 T cells were activated with anti-CD3/CD28 beads and CD8 T cell–derived SNs were incubated with the donor-matched fibroblasts. Gene expression profiles of the fibroblasts were generated and about 4,680 DEGs were observed (Fig. 2 B and Table S2). Very similar to what was seen with the MRC-5 fibroblast cell line, tissue CD8 T cell bystander activation of primary fibroblasts led to the induction of a variety of secondary effector molecules such as *IL6*, *VEGFC*, *CSF1*, and immune regulatory molecules such as *IDO1*, *IL4L1*, *IL18BP*, and *CD274* (PD-L1). These data suggest that our findings are relevant for the interaction of primary tissue CD8 T cells and autologous primary human fibroblasts. To further extend these findings toward epithelial cells, we cocultured epithelial cells (HaCaT) with cell-free SN of influenza-specific T cells cultured on fibroblasts pulsed with varying peptide concentrations and identified >10,000 DEGs with the highest concentration of 100 ng/ml peptide; 6,781 with 10 ng/ml peptide; and 3,593 DEGs with 1 ng/ml peptide versus no peptide (Fig. 2 C and Table S3). Gene set enrichment analysis (GSEA) on both datasets (fibroblasts and epithelial cells) revealed increased IFN signaling in response to stimulation with SNs (Fig. 2 D). These modules included IFN-γ response genes such as *IDO1* and *IFIT3*, which could be induced in a dose-dependent manner by the stimulation of epithelial cells (HaCaT) with SN generated from the three-cell-type system (Fig. 2 E). Moreover, when IFN-γ was inhibited in the three-cell-type system by a blocking antibody, *IDO1* and *IFIT3* were not increased in the epithelial cells (Fig. 2 F), suggesting that IFN-γ signaling plays an important role in the activation of epithelial cells as bystander cells when CD8 T cells undergo antigen-specific activation.

In summary, our findings show that upon antigen-specific TCR activation, CD8 T cells mediate wound healing effects and also govern fibroblast and epithelial cell activation as a bystander effect that potentiates the regenerative potential via the release of tissue remodeling and immune modulatory factors. Thereby, activated CD8 T cells can directly influence their local stromal environment in addition to target cell killing.

### Tissue and blood PD1^+^TIGIT^+^ CD8 T cells are clonally related and express effector molecules

**Figure S2.**
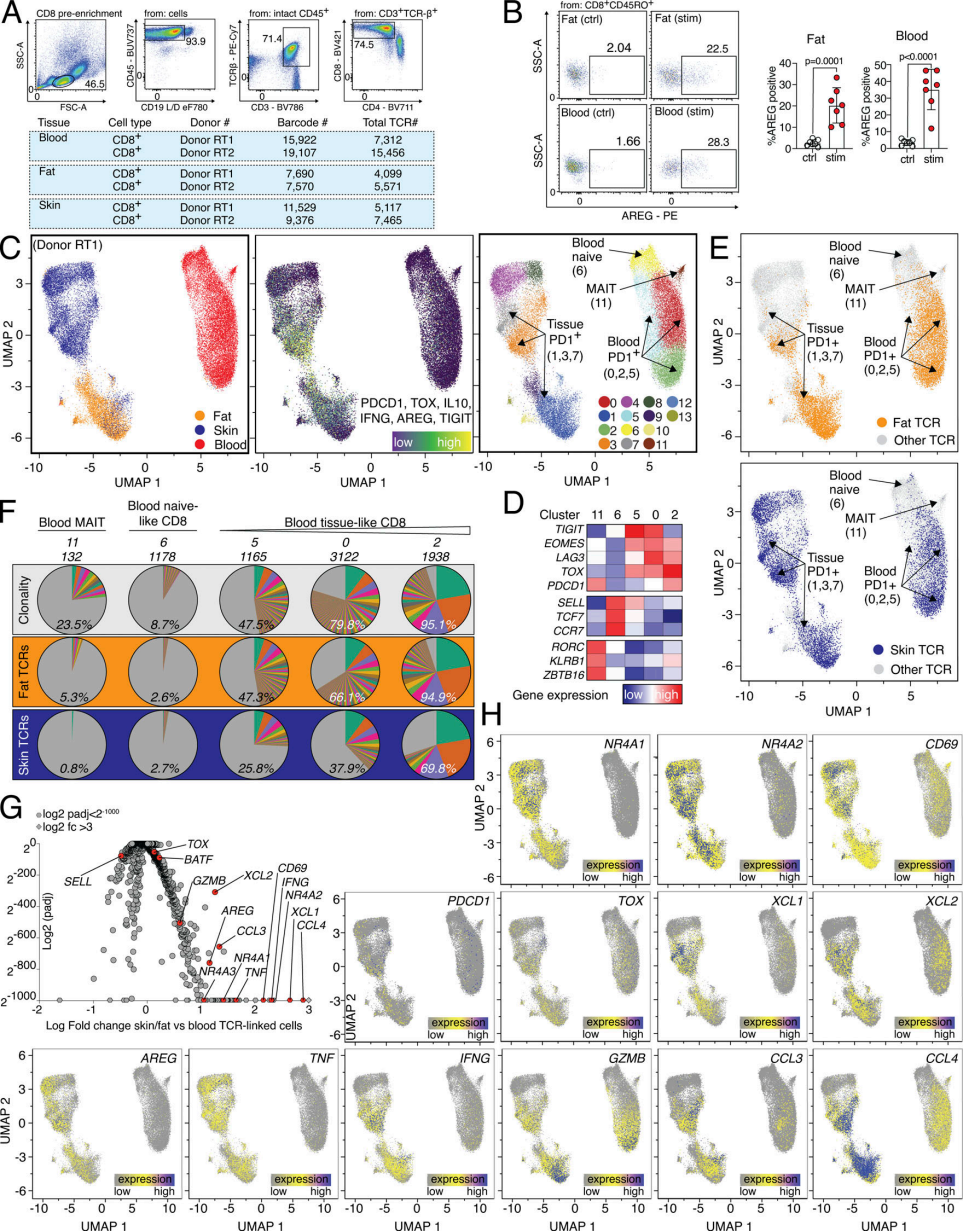
**scRNA/TCR-seq of human CD8 T cells with donor RT1. (A)** Top, sort layout for CD8 pre-enriched immune cells from human blood. Bottom, QC of cells used for combined scRNA/TCR-seq isolated from human blood, fat, and skin. **(B)** Gating and quantification of AREG expression in human fat (left) and blood (right). CD8 T cells after o/n stimulation with TI (Ctrl) or PMA/ionomycin in the presence of TI (stim), *n* = 7. **(C and D)** Left, cells color-coded based on tissue of origin. Middle, expression of gene signature (*PDCD1*, *TOX*, *IL10*, *IFNG*, *AREG*, and *TIGIT*). Right, cells clustered in 12 groups. Annotation of clusters using signature genes shown in (D) and labeled in UMAP. **(E)** Top, TCRs derived from all fat CD8 T cells in cluster 1 (6,381 cells) are highlighted in yellow and displayed in all other clusters. Bottom, TCRs derived from all skin CD8 T cells in clusters 3,7 (8,627 cells) highlighted in blue and displayed in all other clusters. **(F)** Clonality of clusters (top, white), or tracking of fat (middle, orange) or skin (bottom, blue) CD8 T cells in blood-based clusters of the same donor. The percentage indicates the fraction of detected clones among total clones for the donor, with the total number of clones shown above. Each slice represents a clonotype with the angle representing its fraction among all cells in the respective cluster. **(G)** DEGs between TCR-identical cells in clusters 1, 3, 7 and 0, 2, 5, 6, 11. Several genes highlighted in red and labeled, P_adj_ values <2^−1000^ capped at 2^−1000^. Values >3 capped at 3. **(H)** Gene expression of *NR4A1*, *NR4A2*, *CD69*, *TOX*, *XCL1*, *XCL2*, *AREG*, *TNF*, *IFNG*, *GZMB*, *CCL3*, *PDCD1*, and *CCL4* in CD8 T cells from donor RT1. CDR3 sequences are listed in Table S4. All data are derived from two or more independent experiments with an additional donor RT2 shown in Fig. 3.

**Figure 3.**
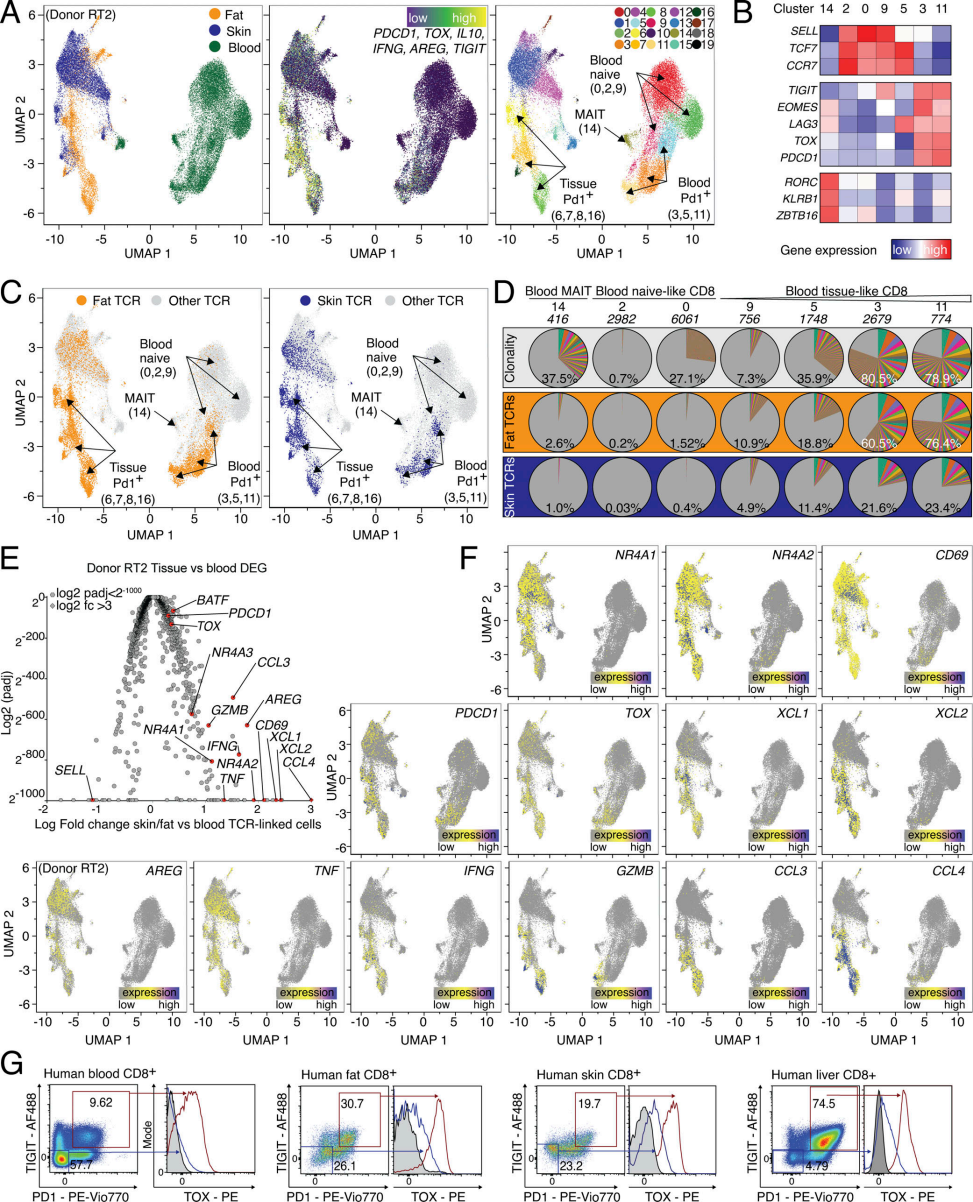
**Human PD1**^**+**^**TIGIT**^**+**^ **CD8 T cells in blood and tissues are clonally related and express effector molecules. (A)** UMAP of scRNA-seq data derived from FACS-sorted CD8 T cell populations of human peripheral blood, skin, and fat of one donor (“Donor RT2”). Left, cells color-coded based on tissue of origin. Middle, expression of gene signature (*PDCD1*, *TOX*, *IL10*, *IFNG*, *AREG*, and *TIGIT*). Right, cells clustered in 20 groups. **(B)** Annotation of clusters using signature genes shown in (B) and labeled in UMAP. Sort info and quality control (QC) in Fig. S2; experimental repeat with second donor (“Donor RT1”) in Fig. S2. **(C)** TCRs derived from fat (left) and skin (right) CD8 T cells in effector cell clusters 6, 7, 8, and 16 (fat: 8,158 cells, skin: 4,573 cells) were highlighted in yellow (fat) or blue (skin) and displayed in all other clusters. **(D)** Clonality of clusters (top, white) or tracking of fat (middle, orange) or skin (bottom, blue) CD8 T cells in blood-based clusters of the same donor. The percentage indicates the fraction of detected clones among the total clones for the donor, with the total number of clones shown above. Each slice represents a clonotype with the angle representing its fraction among all cells in the respective cluster. **(E)** DEGs between TCR-identical cells in clusters 6, 7, 8, 16 and 0, 2, 3, 5, 9, 11, 14, 18. Several genes highlighted in red and labeled, P_adj_ values <2^−1000^ capped at 2^−1000^. Values >3 capped at 3. **(F)** Gene expression of *NR4A1*, *NR4A2*, *CD69*, *TOX*, *XCL1*, *XCL2*, *AREG*, *TNF*, *IFNG*, *GZMB*, *CCL3*, *PDCD1*, and *CCL4* in CD8 T cells from donor RT2. CDR3 sequences are listed in Table S4. **(G)** Expression of intracellular TOX in PD1^+^TIGIT^+^ CD8 T cells from human blood, fat, skin, and liver tissue. All data are derived from two or more independent experiments with the indicated number of human donors.

To determine whether CD8 T cell subsets with regenerative potential, which can be identified by spontaneous expression of the EGFR-ligand AREG, pre-exist in human tissues or blood, we obtained peripheral blood, skin, and subcutaneous adipose tissue from patients undergoing abdominal wall or abdominoplasty surgery. We isolated CD8 T cells using FACS and performed combined single-cell gene expression and TCR-sequencing analysis (scRNA/TCR-seq; Fig. S2 A). Since all three samples were derived from the same individual, this strategy enabled us to measure clonal relationships between blood and tissue-resident T cells (Delacher et al., 2021) and to directly compare the gene expression of tissue-located and circulating CD8 T cells with the same TCR specificity. First, all CD8 T cells from blood and tissue were displayed as Uniform Manifold Approximation and Projection for Dimension Reduction (UMAP, Fig. 3 A, left panel). To identify tissue CD8 T cells with an effector program, a gene expression signature (tissue CD8 T cell signature) was used, including *PDCD1*, *TIGIT*, and *TOX* (Fig. 3 A, middle panel), which identified tissue and blood PD1^+^ CD8 T cells in clusters 6, 7, 8, and 16 (skin and fat tissue) and 3, 5, and 11 (blood, Fig. 3 A, right panel and Fig. 3 B). Other cell populations, such as mucosal-associated invariant T cells or naive CD8 T cells were identified based on lineage marker expression (Fig. 3, A and B). To follow the clonal relation, TCR α and β chain sequences of CD8 T cells from the tissue (fat or skin) *PDCD1*^*+*^*TIGIT*^*+*^*TOX*^*+*^ clusters (6, 7, 8, and 16) were extracted and used to track TCR clones shared between tissue and blood (Fig. 3, C and D). In this tracking, blood CD8 clusters with high gene expression similarity to the tissue CD8 signature corresponded with increased frequencies of shared TCR clones: while blood-based naive CD8 cluster 2 had only about 0.2% overlap with fat-resident CD8 TCRs, blood clusters 3 and 11 shared 60.5% and 76.4% of TCRs with fat-resident CD8. Analogously, while skin-resident CD8 clusters only shared about 0.03% of clones with the blood naive CD8 cluster 2, they shared 21.6% and 23.4% of clones with blood clusters 3 and 11, respectively (Fig. 3 D). The difference between the frequency of fat-associated TCR clones (>60%) and skin-associated TCR clones (about 20%) found in the blood compartment is interesting and needs further evaluation.

To further characterize CD8 T cells with shared TCR repertoires between blood and tissue, we compared skin and fat tissue-resident CD8 T cells (clusters 6, 7, 8, and 16) with blood CD8 T cells expressing the same TCR sequences and identified 2,230 differential expressed genes (Padj < 0.05, Fig. 3 E). In this comparison, CD8 effector/tissue-related genes, such as *BATF*, *PDCD1*, *CD69*, and *TOX*, as well as genes related to TCR signaling (*NR4A1*, *NR4A2*, and *NR4A3*) were higher expressed in tissue-resident CD8 T cells compared with blood CD8 T cells despite sharing the same TCR clones. In addition, various effector molecules and chemokine ligands were overexpressed in tissue-resident CD8 T cells, including *GZMB*, *CCL3*, *CCL4*, *XCL1*, and *XCL2* (Fig. 3, E and F). Finally, we also detected the regeneration-related molecules *AREG*, *TNF*, and *IFNG* in tissue CD8 T cells, providing a hint toward a potential regenerative function of CD8 T cells also in human tissues. Interestingly, *AREG* expression was significantly higher in tissue-resident CD8 T cells even when compared with blood CD8 T cells that shared the same TCRs, suggesting that processes within the tissue, such as antigen-specific activation, lead to spontaneous AREG expression (Fig. 3, E and F). To confirm that human tissue CD8 T cells are indeed able to produce AREG, we isolated blood and tissue CD8 T cells, stimulated them in the presence of protein transport inhibitors, and confirmed AREG protein expression (Fig. S2 B).

The clonal relationship between blood and tissue PD1^+^TIGIT^+^ CD8 T cells was confirmed with a second donor where we identified PD1^+^TIGIT^+^ CD8 T cells, tracked fat and skin-derived CD8 TCR clones in the blood of the same donor, and calculated the TCR overlap (Fig. S2, C–H): blood CD8 clusters with higher similarity for the tissue signature again corresponded with increased frequencies of shared TCR clones, e.g., blood clusters 0 and 2 shared 66.1% and 94.9% of TCRs with fat-resident CD8 T cells, and 37.9% and 69.8% of TCRs with skin-resident CD8 T cells, respectively (Fig. S2, C–F). Gene expression comparisons between the same TCR clones located in tissues versus blood revealed 1,346 DEGs and again included activation-related genes, effector molecules, and chemokine ligands, as well as *AREG* in tissue-located PD1^+^TIGIT^+^ CD8 T cells (Fig. S2, G and H), very similar to the findings of the donor characterized in Fig. 3. In summary, in tissues and blood of patients undergoing abdominal wall or abdominoplasty surgery, we identified a CD8 effector T cell population with clonally related TCRs and expression of *PD1*, *TOX*, and *TIGIT*, with high expression of tissue-residency markers (e.g., *CD69*), effector molecules (*IFNG*, *GZMB*, *CCL3*, *CCL4*), and tissue-regenerative molecules (*AREG*, *TNF*). Based on this, we stained human fat, skin, liver, and blood CD8 T cells with antibodies for TIGIT and PD1 and confirmed on protein level that a TIGIT^+^PD1^+^ CD8 population coexpressing TOX is present in all organs (Fig. 3 G).

Taken together, gene expression, TCR clonality, and protein expression indicate a relationship between blood and tissue PD1^+^TIGIT^+^ CD8 T cells, which express genes related to effector function and regeneration, including AREG, in healthy tissues.

### Activated CD8 T cells can produce AREG and promote wound healing

**Figure 4.**
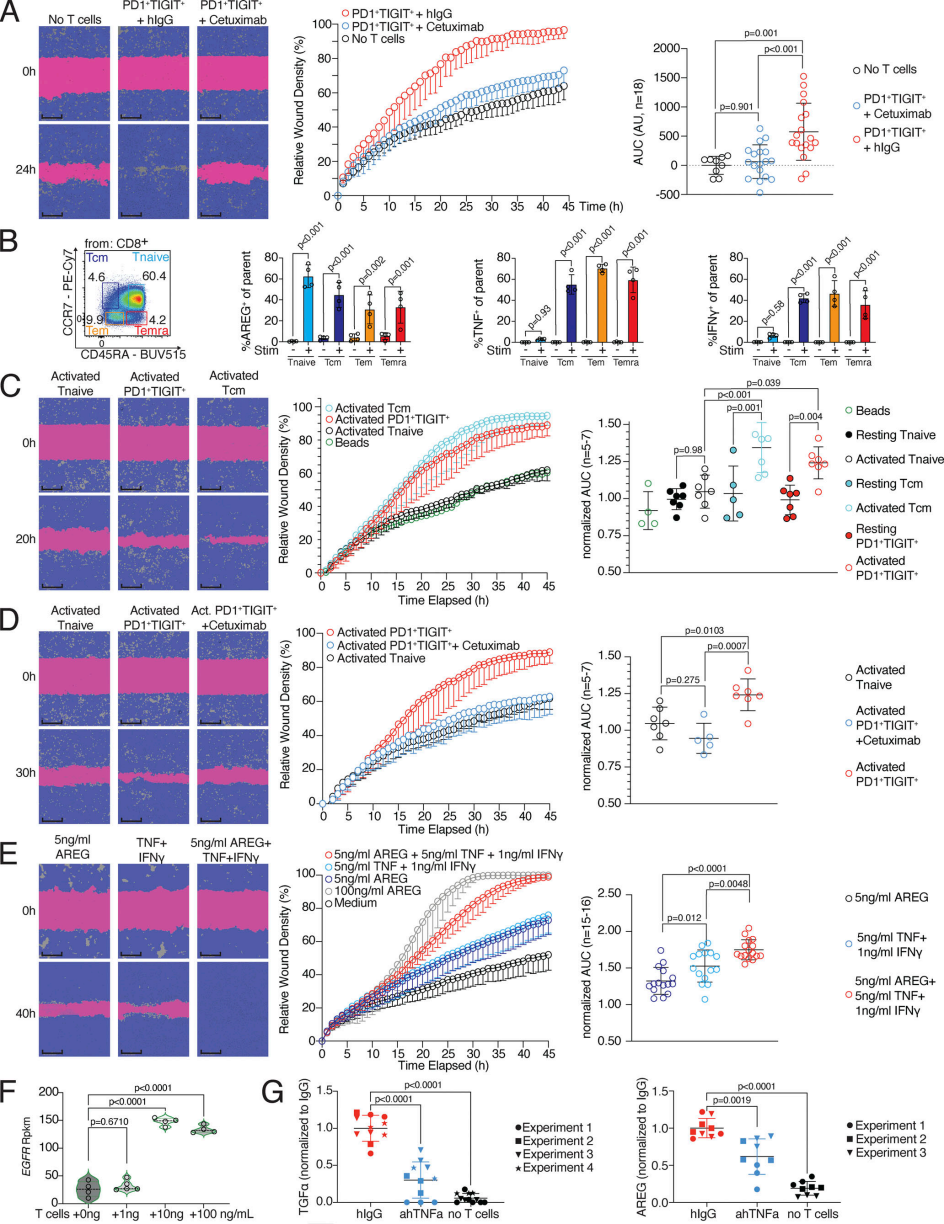
**Subsets of CD8 T cells can instruct wound healing. (A)** Wound healing assay with SNs of human blood-derived CD45RO^+^CD45RA^−^PD1^+^TIGIT^+^ CD8 T cells, in vitro activated by anti-CD3/CD28 beads in the presence of MRC-5 cells, treated with either human IgG or Cetuximab, representative example. Statistical verification across experiments using background-subtracted AUC (*n* = 6, one-way ANOVA). More individual donors in Fig. S3. Scale bars = 400 µm; enhanced for improved visibility. **(B)** Identification of Tnaive (CD45RA^+^CCR7^+^), Temra (CD45RA^+^CCR7^−^), Tem (CD45RA^−^CCR7^−^), and Tcm (CD45RA^−^CCR7^+^) in human blood, followed by intracellular flow cytometry to detect AREG, TNF, and IFN-γ following 4 h incubation in the presence of PMA/ionomycin and transport inhibitors (Stim+) or transport inhibitor only (Stim−, one-way ANOVA with *n* = 4). Additional gating and controls in Fig. S3. **(C)** Human blood-derived Tnaive, Tcm, and CD45RA^+/−^CCR7^−^PD1^+^TIGIT^+^ CD8 T cells were either in vitro cultivated (resting) or in vitro cultivated and activated by anti-CD3/CD28 beads (activated), followed by collection of cell-free SN, which was then used in a wound healing assay with HaCaT cells. Representative images show wound density at 0 and 20 h following wounding and application of cell-free SNs. Statistical verification using normalized AUC (*n* = 5–7, one-way ANOVA). Scale bars = 400 µm; enhanced for improved visibility. **(D)** Wound healing assay with SNs of human blood-derived and in vitro activated and co-cultured PD1^+^TIGIT^+^ or Tnaive CD8 T cells, treated with Cetuximab during wound healing assay (*n* = 6, unpaired *t* test), representative images show wound density at 0 and 30 h following wounding and application of SNs. Statistical verification using normalized AUC (*n* = 5–7, one-way ANOVA). Scale bars = 400 µm; enhanced for improved visibility. **(E)** Wound healing assay with human recombinant AREG (100 or 5 ng/ml), TNF (5 ng/ml), and IFN-γ (1 ng/ml), representative images of wound density 0 and 40 h after initial wounding shown. Statistical verification using normalized AUC (*n* = 15–16, one-way ANOVA). Scale bars = 400 µm; enhanced for improved visibility. **(F)** EGFR expression in HaCaT cells stimulated o/n with SN generated in the three-cell-type system. Results derived from sequencing data from Fig. 2 C. **(G)** TGFα (left) and AREG (right) level in SN of three-cell-type system as described in Fig. 1 A in the presence of hIgG, anti-hTNF, or no T cells. Cytokine levels determined by ELISA. All data were derived from several independent experiments with the indicated number of donors.

**Figure S3.**
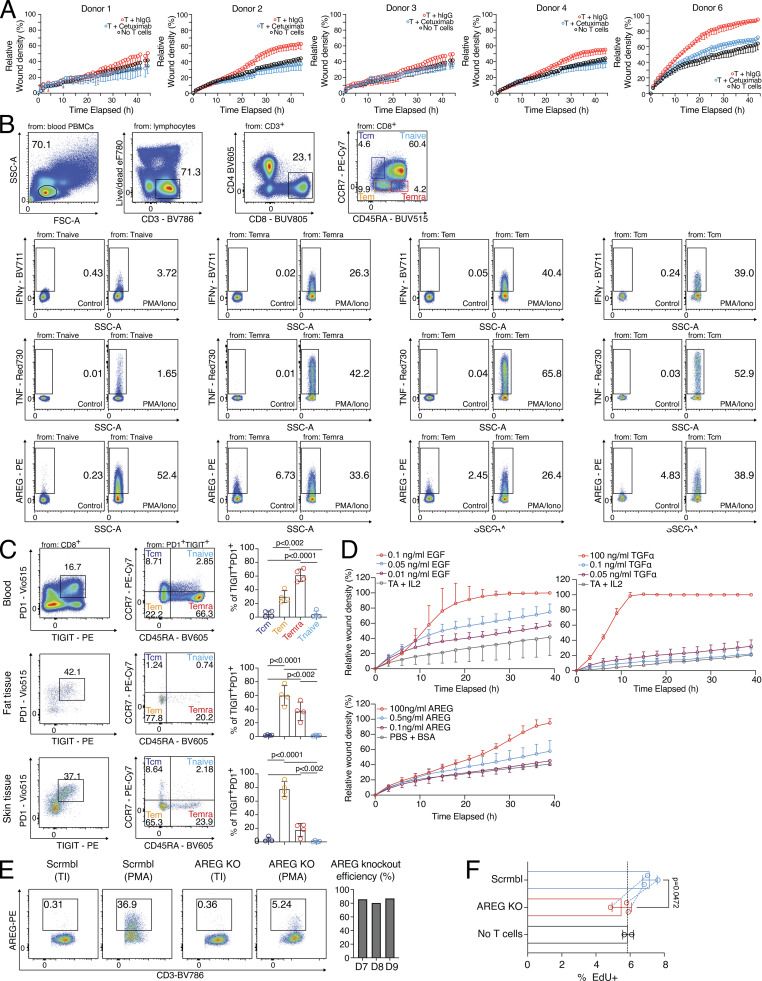
**Cytokine expression and wound healing potential of blood-derived CD8 T cells. (A)** Wound healing assay with SNs of human blood-derived and in vitro activated and cocultured PD1^+^TIGIT^+^ CD8 T cells (T = T cells), treated with either human IgG or Cetuximab during wound healing assay; several donors. **(B)** Gating to identify Tnaive (CD45RA^+^CCR7^+^), Temra (CD45RA^+^CCR7^−^), Tem (CD45RA^−^CCR7^−^), and Tcm (CD45RA^−^CCR7^+^) in human blood, followed by intracellular flow cytometry to detect human AREG, TNF, and IFN-γ following 4 h incubation in the presence of PMA/ionomycin and transport inhibitors or transport inhibitor only. **(C)** Gating to identify Tnaive (CD45RA^+^CCR7^+^), Temra (CD45RA^+^CCR7^−^), Tem (CD45RA^−^CCR7^−^), and Tcm (CD45RA^−^CCR7^+^) in PD1^+^TIGIT^+^ CD8 T cells from human blood, fat, and skin tissue. Statistics to the right (one-way ANOVA, *n* = 4). **(D)** Wound healing assay with varying concentrations of recombinant human EGF, TGFα, or AREG. **(E)** AREG CRISPR KO efficiency. Left, exemplary flow cytometry plots depicting AREG expression; right, quantification. **(F)** EdU incorporation in HaCaT cells treated o/n with SN from scrmbl CD8 T cells, AREG knockout CD8 T cells or no T cell control (*n* = 3, Student’s *t* test). All data were derived from experiments with several independent donors.

In the next step, we functionally addressed the tissue regenerative potential of primary PD1^+^TIGIT^+^ CD8 T cells. Since CD8 T cells from tissues are difficult to obtain in large numbers, we sorted the clonally related PD1^+^TIGIT^+^ CD8 T cell population from blood, restimulated it with anti-CD3/CD28 beads to promote TCR activation, and cultured these cells in the presence of fibroblasts (MRC-5) to simulate tissue conditions. Cell-free SN was collected after 16 h and evaluated in a wound healing assay with the keratinocyte cell line (HaCaT) in a live cell imaging system. In this assay, SNs showed reparative abilities, and inhibiting the EGFR signaling pathway by Cetuximab abrogated these reparative abilities (Fig. 4 A and Fig. S3 A), as observed previously with influenza-specific CD8 T cells (Fig. 1). To understand how widely the regeneration-related molecules AREG, TNF, and IFN-γ are expressed in human blood CD8 T cells after activation, we analyzed CD8 T cell subpopulations from Peripheral blood mononuclear cells (PBMCs, T effector memory [Tem, CD45RA^−^CCR7^−^], T central memory [Tcm, CD45RA^−^CCR7^+^], terminally differentiated memory T [Temra, CD45RA^+^CCR7^−^], and naive T cells [CD45RA^+^CCR7^+^], as defined in Sallusto et al. [1999]). AREG was produced upon PMA/ionomycin stimulation in all populations, whereas TNF and IFN-γ were expressed in all but the naive CD8 T cell fraction after activation (Fig. 4 B and Fig. S3 B). A closer look at the tissue and blood PD1^+^TIGIT^+^ CD8 T cell populations revealed that they were mainly CCR7^−^ (Fig. S3 C), mostly resembling the Tem and Temra phenotypes. Next, to address the potency of PD1^+^TIGIT^+^, Tcm, and naive CD8 T cells to induce tissue remodeling in vitro, we sorted all populations by FACS and restimulated the cells with anti-CD3/CD28 beads to induce TCR activation, this time in the absence of fibroblasts. The SN derived from activated PD1^+^TIGIT^+^ CD8 T cells and activated Tcm showed comparable reparative abilities in the in vitro wound healing assay, while activated naive CD8 T cells were unable to promote this function (Fig. 4 C). SNs from cells without TCR activation (resting) had no reparative function (Fig. 4 C). Blocking EGFR signaling with Cetuximab again abrogated tissue repair functionalities in the in vitro wound healing assay using SN from activated PD1^+^TIGIT^+^ CD8 T cells (Fig. 4 D). To directly prove the involvement of the EGFR ligands in our wound healing assay, we tested different recombinant ligands. Our results show that varying concentrations of AREG, TGFα, or EGF induced increased wound healing in a dose-dependent manner (Fig. S3 D).

To further prove the functional relevance of AREG in the SN generated from CD8^+^ T cells, we knocked out AREG in CD8 T cells using the CRISPR-Cas9 technology, achieving a knockout efficiency of ∼80% (Fig. S3 E). We then stimulated epithelial (HaCaT) cells with cell-free SNs generated from these AREG-KO CD8 or scrambled control guide CD8 T cells for 18 h and measured EdU incorporation after 1 h of EdU incubation as a measure of induced cell proliferation in the epithelial cells. We observed significantly decreased EdU incorporation in epithelial cells stimulated with the AREG-KO SN compared to cells stimulated with the scrambled control guide CD8 T cell SN, indicating that AREG released by CD8 T cells is at least partially responsible for the wound healing capacity of the SN (Fig. S3 F).

The failure of antigen-naive CD8 T cells to promote regeneration despite their capacity to produce AREG suggests that AREG production by CD8 T cells alone is not sufficient to mediate regenerative functions in our system. Instead, the regenerative function of AREG could require joint action with other effector molecules only produced by activated PD1^+^TIGIT^+^ or effector CD8 T cells. To further address whether the reparative function of AREG can be enhanced with other molecules secreted by activated PD1^+^TIGIT^+^ CD8 T cells, we performed the wound healing assay with two concentrations of recombinant AREG: high-dose (100 ng/ml) and low-dose (5 ng/ml). The AREG high-dose condition efficiently promoted the closure of the wound, while the AREG low-dose condition was less efficient in promoting wound closure (Fig. 4 E). TNF and IFN-γ are both associated with tissue remodeling (Chen et al., 2007; Cheng et al., 2008; Leppkes et al., 2014), and both cytokines were highly secreted by PD1^+^TIGIT^+^ CD8 T cells. Therefore, we added recombinant TNF and IFN-γ to the AREG low-dose condition and performed the wound healing assay again. The data demonstrated that the reparative ability of low-dose AREG was substantially increased by adding IFN-γ and TNF (Fig. 4 E). Furthermore, when analyzing epithelial cells (HaCaT) stimulated with SN generated in the three-cell-type system described in Fig. 1 A, we found the EGFR to be significantly higher expressed in SN-stimulated HaCaT cells (Fig. 4 F), indicating that some of the observed increase in wound healing capacity may be due to increased EGFR signaling, promoted by CD8 T cells. This was further substantiated by the observation that when TNF was inhibited during SN generation, significantly lower levels of the EGFR ligands TGFα and AREG were detected in the SN (Fig. 4 G), suggesting that TNF released by CD8 T cells can induce the expression of EGFR ligands by epithelial cells.

In summary, our findings indicate that activated PD1^+^TIGIT^+^ and effector CD8 T cells have the potential to induce tissue regeneration after TCR stimulation. Furthermore, TNF and IFN-γ can sensitize epithelial cells for AREG-mediated regenerative potential. The sensitization effect of CD8 T cells can be explained by induced expression of the EGFR and EGF-ligands by epithelial cells.

### Human CD8 tumor-infiltrating lymphocytes (TILs) and CAR T cells have both cytotoxic and tissue regeneration potential

**Figure S4.**
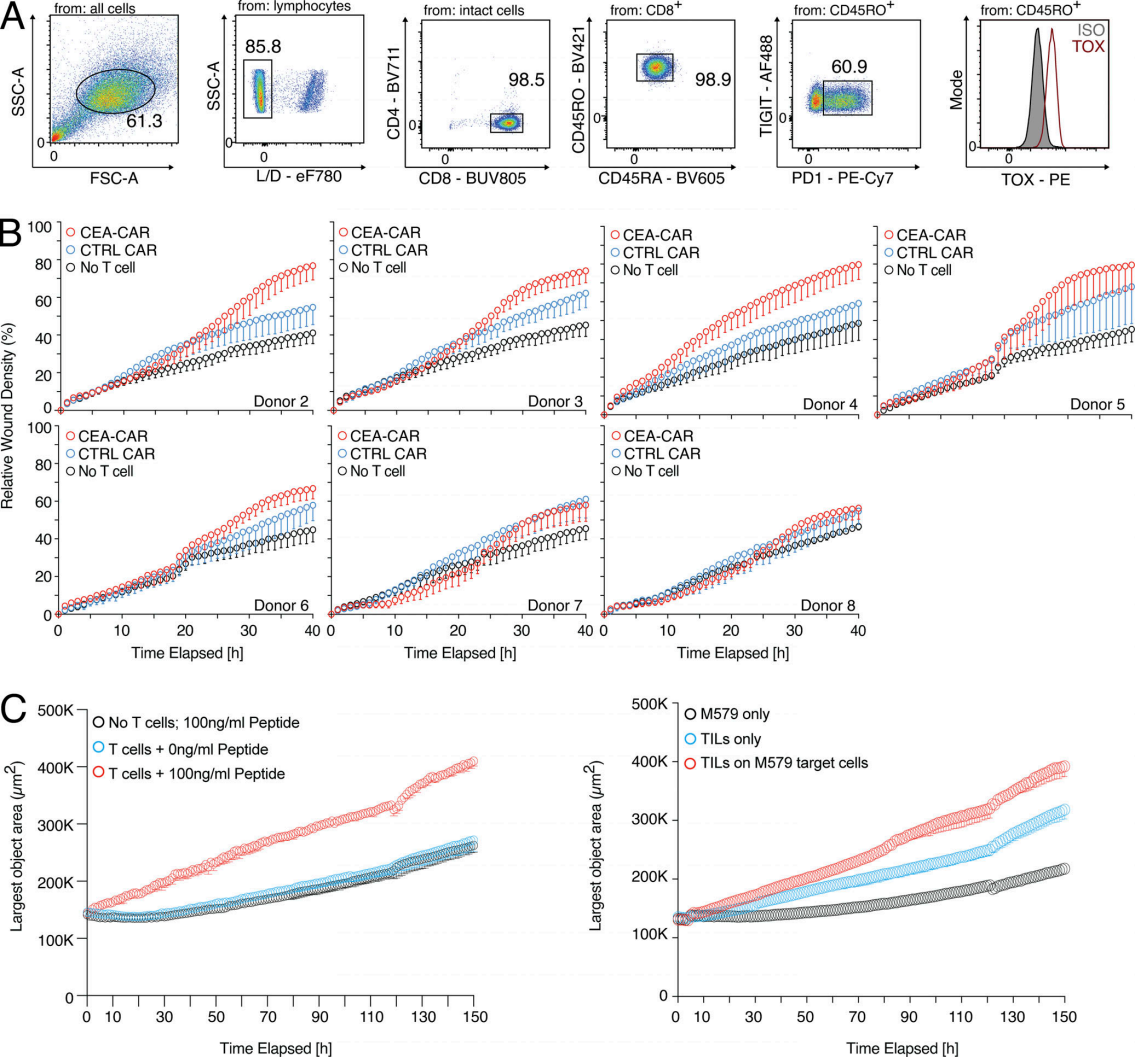
**Human CD8 T cells can promote tumor growth in vitro. (A)** Expression of TOX in TILs. **(B)** Wound healing assay with SN derived from CEA-CAR-transgenic CD8 T cells versus Ctrl-CAR-transgenic CD8 T cells or no T cells; individual donors are shown. **(C)** Spheroid assay using cell-free SN from influenza-specific CD8 T cells and varying amounts of pulsed peptides on MRC-5 and HaCaT cells seeded in a 30:70 ratio (left) or tumor-TIL coculture (right); experimental repeat of Fig. 6, B and C.

**Figure 5.**
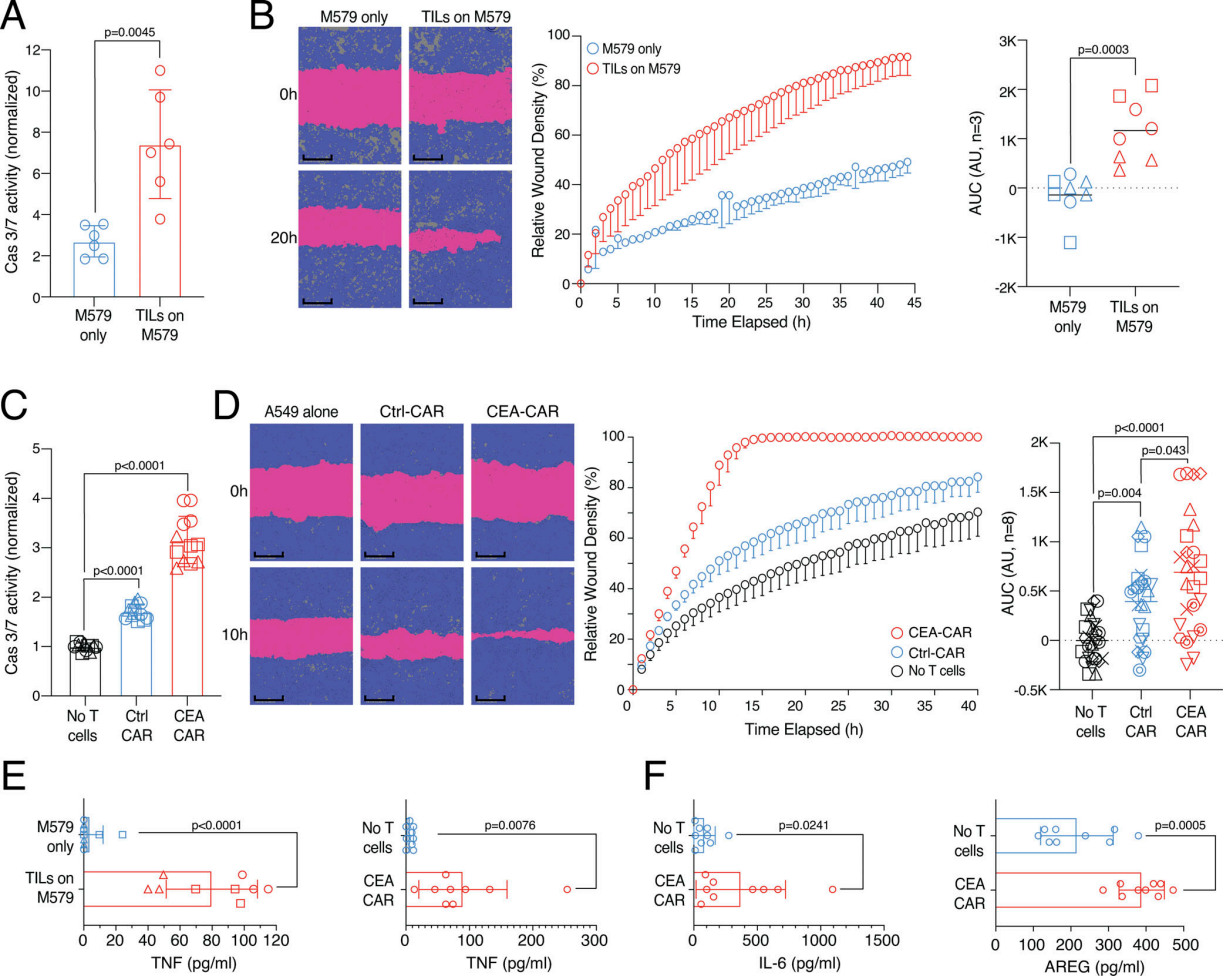
**Human CD8 TILs and CAR T cells can support tumor cell killing and wound healing in vitro. (A)** TIL-target cell coculture with killing activity of TILs measured via green fluorescence (Cas 3/7, *n* = 3, unpaired *t* test). **(B)** Wound healing assay with HaCaT cells using SN derived from TIL-target cell coculture or M579 only. Statistical verification across experiments using background-subtracted AUC (*n* = 3, unpaired *t* test of AUC, symbols indicate individual experiments). Scale bars = 400 µm; enhanced for improved visibility. **(C)** CEA-CAR-transgenic CD8 T cells, Ctrl-CAR-transgenic CD8 T cells, or no T cells were cocultured with CEA-expressing A549 target cells. Killing activity of CAR T cells was measured via green fluorescence (Cas 3/7, *n* = 3, unpaired *t* test, symbols indicate individual experiments). **(D)** Wound healing assay with SN derived from CEA-CAR-transgenic CD8 T cells versus Ctrl-CAR-transgenic CD8 T cells or no T cells. Representative example; more donors in Fig. S4. Statistical verification across experiments using background-subtracted AUC (*n* = 8, one-way ANOVA of AUC, symbols indicate individual experiments). Scale bars = 400 µm; enhanced for improved visibility. **(E)** TNF in TIL-target cell co-culture (left, *n* = 3, unpaired *t* test, symbols indicate individual experiments) or CEA-CAR-transgenic CD8 T cell coculture (right, *n* = 9, paired *t* test). **(F)** IL-6 (left) and AREG (right) in CEA-CAR-transgenic CD8 T cell co-culture (*n* = 9, paired *t* test). All data were derived from two or more independent experiments with the indicated number of replicates.

After demonstrating the regenerative potential of blood PD1^+^TIGIT^+^ CD8 T cells (Fig. 4), we checked whether the regenerative effector program is also active in CD8 T cells used for anticancer therapy, such as TILs or CAR T cells. To investigate this, we established a second antigen-specific in vitro system with melanoma tumor cells (M579) and autologous TOX-positive CD8 TILs (Fig. S4 A). This system showed cytotoxicity against the autologous M579 tumor cells (Fig. 5 A), alongside increased wound healing capacity of the coculture-derived SN (Fig. 5 B), indicating that wound healing and target killing are also both effector mechanisms in the tumor cell–TIL interaction in vitro.

CD8 CAR T cells are currently heavily investigated to combat cancer. To study whether these anticancer effector cells also possess tissue regenerative abilities, CD8 T cells of several donors were transduced with a CAR against the carcino-embryonic-antigen (CEA) or with a control plasmid (Ctrl) and cocultured with CEA-expressing lung epithelial cells (A549). In this assay, antigen-specific target cell killing was observed (Fig. 5 C) and SN derived from anti-CEA-CAR CD8 T cells showed enhanced wound healing capacity compared with the Ctrl-CAR (Fig. 5 D and individual donors in Fig. S4 B). In both the CD8 TIL-tumor coculture and the CD8 CEA-CAR coculture, increased levels of TNF were observed (Fig. 5 E), as well as increased IL-6 and AREG levels in the anti-CEA-CAR system (Fig. 5 F). These findings indicate that effector CD8 T cells, including anti-cancer CD8 T cells (TILs, CARs), can promote both wound healing and tumor target cell killing.

### The CD8 T cell remodeling potential is linked to increased tumor spheroid growth

**Figure 6.**
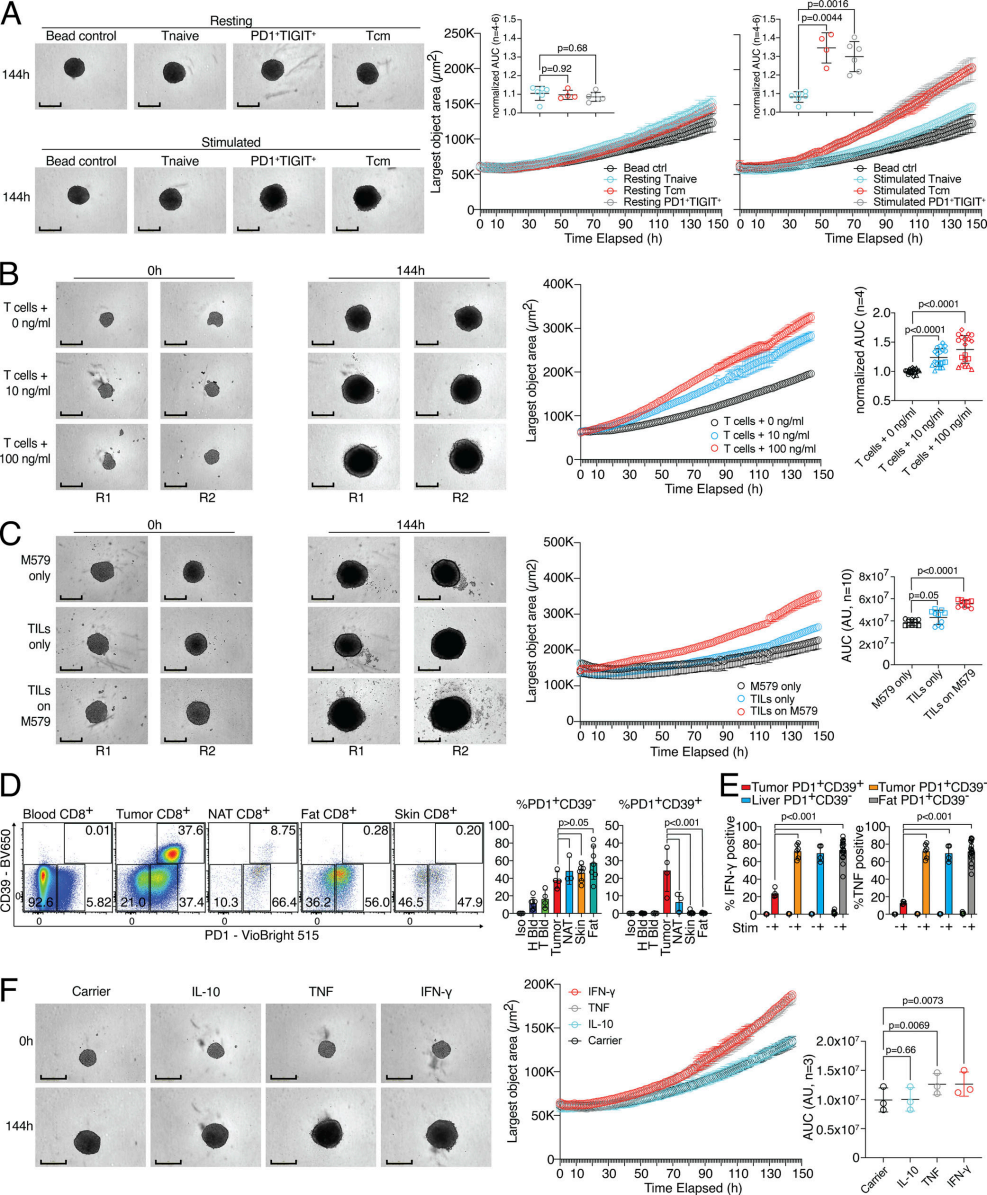
**Human CD8 T effector cells can support tumor spheroid growth. (A)** CD8 Tnaive, Tcm, and PD1^+^TIGIT^+^ were isolated from human blood and ex vivo cultivated (resting) or ex vivo cultivated and activated (stimulated). Cell-free SNs were used in a spheroid assay, and spheroid growth was observed over time. Left, representative images with bead control, unstimulated, or stimulated Tnaive, Tcm, or PD1^+^TIGIT^+^ CD8 T cells. Right, largest object areas over cultivation time with statistical verification across experiments using normalized AUC (*n* = 4–6, one-way ANOVA). Scale bars = 400 µm; enhanced for improved visibility. **(B)** Spheroid assay using cell-free SN from influenza-specific CD8 T cells and varying amounts of pulsed peptides on MRC-5 and HaCaT cells seeded in a 30:70 ratio with statistical verification across experiments using normalized AUC (*n* = 4, one-way ANOVA of AUC, symbols indicate individual experiments), experimental repeat in Fig. S4. Scale bars = 400 µm; enhanced for improved visibility. **(C)** Spheroid assay using cell-free SN from TIL-target cell coculture with statistical verification across experiments using background-subtracted AUC (*n* = 10, one-way ANOVA of AUC, symbols indicate individual experiments), experimental repeat in Fig. S4. Scale bars = 400 µm; enhanced for improved visibility. **(D)** Expression of CD39 and PD1 in CD8 T cells from tumor patient blood, liver tumor, liver NAT, fat, and skin; statistics to the right (*n* = 3–7, one-way ANOVA). **(E)** Intracellular deposition of IFN-γ and TNF in PMA/ionomycin and TI-stimulated liver tumor, liver NAT, and fat CD8 T cell subpopulations (*n* = 4–17, one-way ANOVA). **(F)** Spheroid assay using recombinant cytokines. Left, representative images with carrier, IL-10, TNF, and IFN-γ. Right, largest object area over cultivation time, with statistical verification across experiments using background-subtracted AUC (*n* = 3, one-way ANOVA of AUC). Scale bars = 400 µm; enhanced for improved visibility. All data are derived from two or more independent experiments with the indicated number of replicates.

To investigate whether the tissue-remodeling ability of the SNs from activated human CD8 T cells also affects tumor growth, we established a 3D tumor spheroid growth assay with human colon carcinoma cells (HCT116). We sorted PD1^+^TIGIT^+^, naive, and Tcm CD8 T cells from human blood and activated the different populations with anti-CD3/CD28 stimulation as before. SN derived from these cell types was applied to tumor spheroid cultures and 3D spheroid object area was measured in an automated fashion over several days in culture. Without TCR stimulation, none of the SNs induced an accelerated tumor spheroid growth over time (Fig. 6 A, top and right panel). In contrast, SNs of TCR-stimulated PD1^+^TIGIT^+^ and Tcm, but not Tnaive, CD8 T cells showed significant growth-promoting abilities (Fig. 6 A, bottom and right panel). To study the tumor spheroid growth-promoting potential of activated human CD8 T cells in an antigen-specific model, SNs of the three-cell-type system (as described in Fig. 1) with 100 ng/ml, 10 ng/ml, and without influenza peptide were tested. SNs of the 100 ng/ml and 10 ng/ml peptide-activated CD8 T cells could significantly increase tumor spheroid growth in a dose-dependent fashion, as compared to T cells with no peptide (Fig. 6 B and Fig. S4 C, left). Additionally, SNs from the tumor-TIL system promoted tumor spheroid growth over time (Fig. 6 C and Fig. S4 C, right).

To further dichotomize between tumor-reactive CD8 T cells, which can become dysfunctional or exhausted (T_EX_) in certain cancer types (Pritykin et al., 2021) and are usually the target of checkpoint blockade therapy, and tissue-resident CD8 T cells, we costained CD39 and PD1 in tissue from liver cancer, normal tissue adjacent to the liver tumor (NAT), and in skin, fat, and blood of patients undergoing abdominal wall or abdominoplasty surgery (Fig. 6 D). Our data indicate that only tumor tissue contained a high fraction of PD1^high^CD39^+^ T_EX_, while all analyzed tumor and non-tumor tissue contained PD1^+^CD39^−^ CD8 T cells. Functional analysis of the different CD8 T cell populations from tumor and healthy tissues showed that fewer than 20% of tumor-derived PD1^+^CD39^+^ T_EX_ produced IFN-γ and TNF, while about 80% of PD1^+^CD39^−^ CD8 T cells from normal human tissue, NAT, and liver tumor tissue produced these cytokines after restimulation (Fig. 6 E). To determine whether effector cytokines produced by these functional tissue PD1^+^CD39^−^ CD8 T cells could promote tumor spheroid growth on their own, we tested recombinant human IFN-γ and TNF in tumor spheroid growth assays (Fig. 6 F). While both IL-10 as an anti-inflammatory cytokine control and a carrier control showed baseline spheroid growth, both IFN-γ and TNF could, individually, promote significant tumor spheroid growth. This highlights a dual role for these effector molecules in supporting target cell killing and tissue remodeling. Together, these data indicate that CD8 effector T cells can support both tumor cell killing and tumor cell growth, a finding that is highly relevant for the design of therapeutic T cell products.

### Tissue regenerative abilities of human CD8 T cells support organoid growth

**Figure 7.**
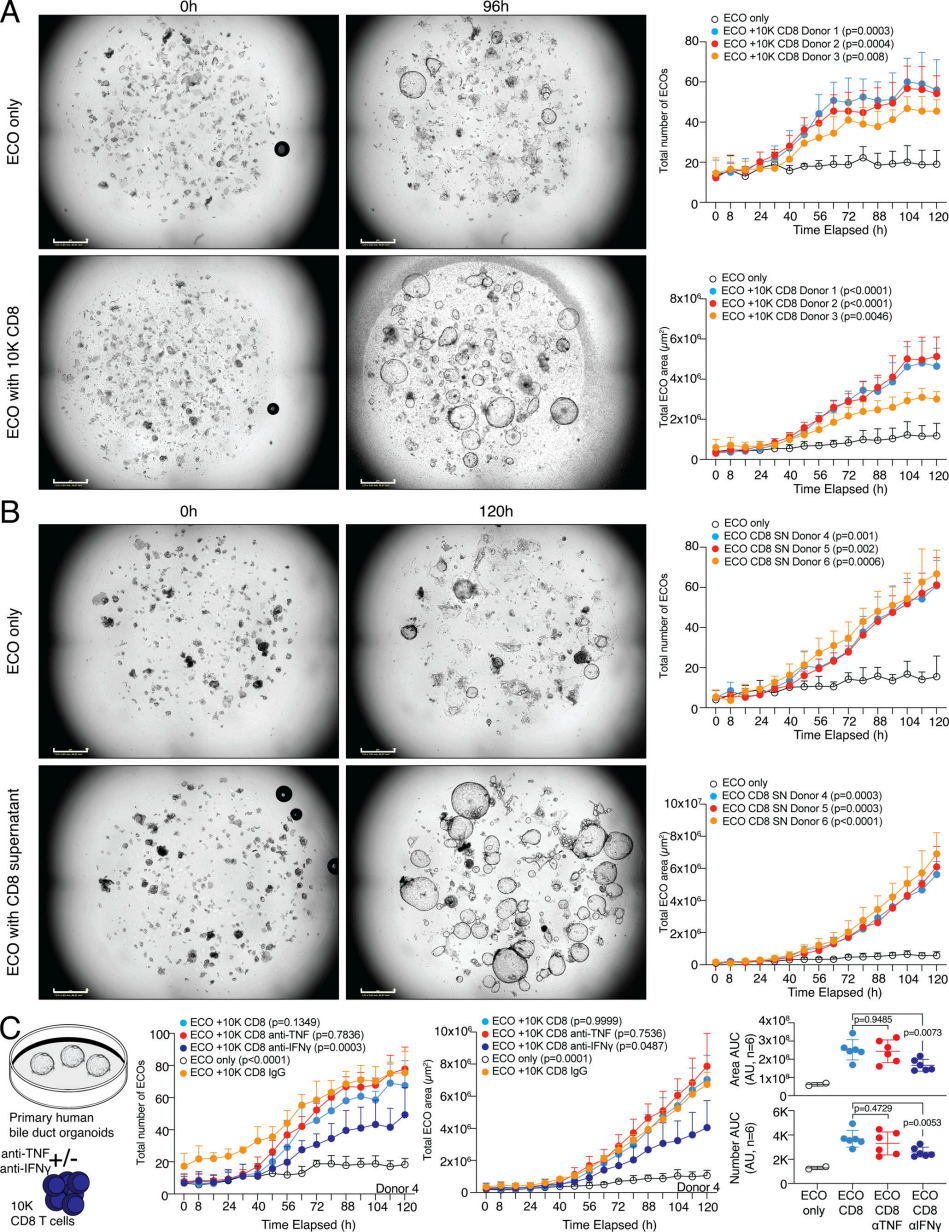
**Human CD8 T effector cells can promote tissue regeneration in organoids. (A)** 20 ECO organoids were dissociated and cultured alone or in coculture with 10,000 CD8 T cells derived from different donors (D1–D3) and organoid growth was observed over time. Left, representative images of organoids. Right, quantification of the number of organoids (top) and total organoid area (bottom) over cultivation time with statistical verification using AUC (*n* = 4 wells, one-way ANOVA, compared to ECO only). Additional individual donors are shown in Fig. S5. Growth kinetics are shown in Videos 1 and 2. Scale bars = 800 µm; enhanced for improved visibility. **(B)** 20 ECO organoids were dissociated and cultured alone or in coculture with cell-free SN of 200,000 activated CD8 T cells derived from different donors (D4–D6) and organoid growth was observed over time. Left, representative images of organoids. Right, quantification of the number of organoids (top) and total organoid area (bottom) over cultivation time with statistical verification using AUC (*n* = 4 wells, one-way ANOVA, compared to only ECO). Additional individual donors are shown in Fig. S5. Growth kinetics are shown in Videos 3 and 4. Scale bars = 800 µm; enhanced for improved visibility. **(C)** 20 ECO organoids were dissociated and cultured alone or in coculture with 10,000 CD8 T cells derived from different donors (D1–D6) in presence or absence of anti-IFN-γ or anti-TNF and organoid growth was observed over time. IgG served as a control. Left, experimental layout. Middle, quantification of the number of organoids and total organoid area over cultivation time from D4 (*n* = 4 wells, one-way ANOVA, compared to IgG). Right, quantification of organoid area and number from all donors of experiment 1 and 2 (D1–D6) combined with statistical verification using AUC from 48 h onwards (*N* = 6 donors, one-way ANOVA, compared with CD8, symbols indicate individual experiments). Additional individual donors from experiment 1 (D1–D3), experiment 2 (D4–D6), and experiment 3 (D9–D11) are shown in Fig. S5. All data derived from two or more independent experiments with the indicated number of replicates and donors.

**Figure S5.**
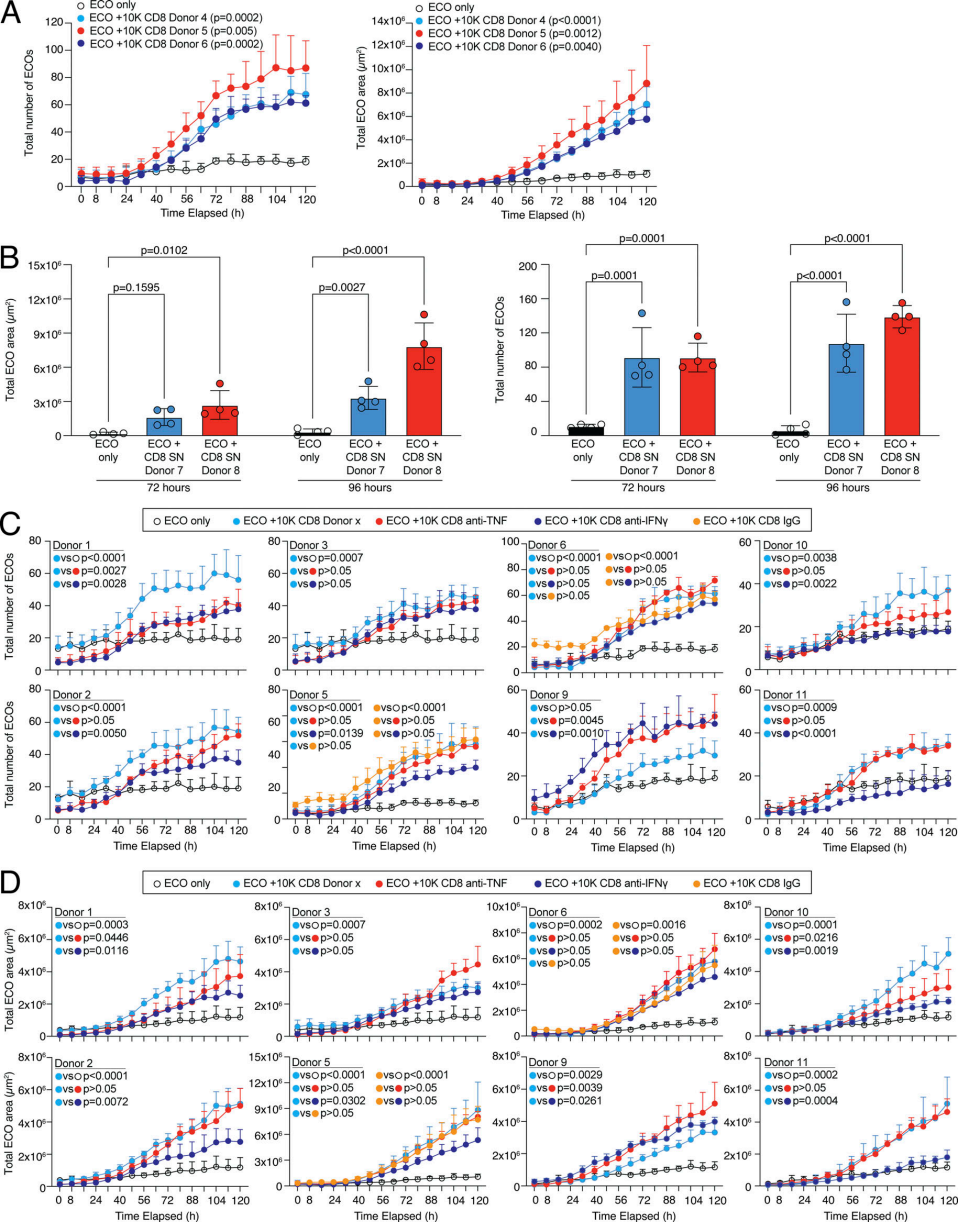
**Human CD8 T cells can promote tissue regeneration in organoids. (A)** 20 ECO organoids were dissociated and cultured alone or in coculture with 10,000 CD8 T cells derived from different donors (D4–D6) and organoid growth was observed over time. Quantification of the number of organoids (left) and total organoid area (right) over cultivation time with statistical verification using AUC (*n* = 4 wells, one-way ANOVA, compared to ECO only). **(B)** 20 ECO organoids were dissociated and cultured alone or in coculture with cell-free SN of 200,000 activated CD8 T cells derived from different donors (D7 and D8) and organoid growth was determined at 72 and 96 h. Quantification of the total organoid area (left) and the number of organoids (right) at indicated time points with statistical verification (*n* = 4 wells, two-way ANOVA, compared to ECO only). **(C and D)** 20 ECO organoids were dissociated and cultured alone or in coculture with 10,000 CD8 T cells derived from different donors (experiment 1 [D1–D3], experiment 2 [D5 and D6], and experiment 3 [D9–D11]) in the presence or absence of anti-IFN-γ or anti-TNF, and organoid growth was observed over time. IgG served as a control. Quantification of organoid number (C) and total organoid area (D) over cultivation time with statistical verification using AUC from 48 h onwards (*n* = 4 wells, one-way ANOVA, compared to CD8 for D1–D3 and D9–D11, and compared to CD8 or IgG for D5 and D6, as depicted).

To address whether human CD8 T cells can also promote tissue regeneration in a more complex stem cell–driven organoid formation model, we made use of an assay system with primary human bile duct organoids, also referred to as extrahepatic cholangiocyte organoid (ECO) cultures (Huch et al., 2015; Sampaziotis et al., 2017; Verstegen et al., 2020). 20 individual ECOs, which contain tissue stem cells important for organoid regeneration, were dissociated and the ability to establish and grow new organoids in the presence or absence of 10,000 TCR-activated human CD8 T cells was monitored in an automated image acquisition system for several days. TCR-activated CD8 T cells from the blood of several donors were tested for their ability to support organoid formation and growth. Cocultures with CD8 T cells showed a clear and highly significant advantage in organoid formation and growth, both recapitulated in the total number of newly formed organoids and the total area occupied by organoids per well (Fig. 7 A and Fig. S5 A; and Videos 1 and 2). To study if this regenerative effect is mediated by soluble factors released by the activated CD8 T cells, the assay was modified and instead of CD8 T cells, cell-free SN of activated CD8 T cells was added to the organoid culture. Organoid formation and growth were strongly promoted by the SN of CD8 T cells, indicating soluble factors supporting organoid growth (Fig. 7 B and Fig. S5 B; and Videos 3 and 4). To address which factors that are released by CD8 T cells could drive this organoid regenerative ability, we blocked IFN-γ and TNF in the cocultures of CD8 T cells and organoids. Our data showed that blocking IFN-γ significantly interfered with CD8-driven organoid formation, which was repeatedly observed in several donors (Fig. 7 C; and Fig. S5, C and D). These data show that activated human CD8 T cells can support tissue regenerative functions in primary stem cell–driven organoids.

**Video 1.** **Organoid growth from** Fig. 7 A **in the control group “ECO only” over time (frame rate 2 pic/second).**

**Video 2.** **Organoid growth from** Fig. 7 A **in the coculture of “ECOs and 10k CD8 T cells” over time (frame rate 2 pic/second).**

**Video 3.** **Organoid growth from** Fig. 7 B **in the control group “ECO only” over time (frame rate 2 pic/second).**

**Video 4.** **Organoid growth from** Fig. 7 B **in the culture of “ECO with CD8 SN” over time (frame rate 2 pic/second).**

## Discussion

In this study, we investigated human tissue and blood CD8 T cells using single-cell transcriptional profiling as well as different tissue remodeling assay systems. T cell receptor sequencing revealed that PD1^+^TIGIT^+^ CD8 T cells in skin and fat of human donors had a very high clonal overlap with blood PD1^+^TIGIT^+^ CD8 T cells. These data are in agreement with a recently published study, which found that the top 10 TCR clones of different memory CD8 T cell populations cover a high fraction of the total TCR repertoire across different memory populations and different organs, e.g., Tem and tissue-resident memory (Trm) cells (Miron et al., 2021). Functional analysis using wound healing, organoid, and 3D tumor spheroid assays identified opposing effector functions. In addition to the well-studied cytotoxic potential of human CD8 T cells, activation of CD8 T cells also led to the production of molecules associated with wound healing and to a bystander activation of fibroblasts and epithelial cells, promoting primary organoid growth and accelerating tumor spheroid growth. Using our scRNA-seq data, we found that PD1^+^TIGIT^+^ CD8 T cells present in healthy tissues such as skin and fat shared signatures with pre-exhaustion CD8 T cells and expressed tissue-resident markers such as CD69, effector molecules (*GZMB*, *CCL3*, and *CCL4*), and tissue-regenerative molecules (*AREG*, *TNF*, and *IFNG*). Our data show that these cells closely resemble previously described Trm cells (Gray and Farber, 2022; Poon et al., 2023) and suggest a tissue regenerative potential of this population. This is supported by a recent study using a multiomics approach which reported on human CD8 T cell subsets identified in different cancer types and specifically enriched in glioblastoma with wound healing signatures (Naulaerts et al., 2023). In this study, anti-PD-1 blockade was associated with an increase in these wound healing signatures (Naulaerts et al., 2023), emphasizing the importance of better understanding the tissue regenerative potential of human CD8 T cells. The here-described human CD8 T cell regenerative capacities might explain the observation that preactivated effector-like CD8 T cells contribute to liver pathology under chronic NASH conditions and promote malignant transformation and tumor growth rather than exert antitumor effector functions (Dudek et al., 2021; Pfister et al., 2021). While these published data suggested that TNF is an important factor to promote fibrosis in the NASH model, our data add IFN-γ and AREG as relevant molecules readily produced by human CD8 T cells and promoting tissue remodeling. Also in the murine system, commensal-specific CD8 T cells in the skin have been reported to express a defined gene signature that is characterized by the expression of effector genes together with immunoregulatory and tissue-repair genes, which could support accelerated skin wound closure (Linehan et al., 2018). All these findings are especially important since CD8 T cells are used to combat cancer by adoptive transfer of tumor antigen-specific CAR T cells or TCR-transgenic T cells (Larson and Maus, 2021). Our findings now indicate that the cytotoxic effector program is tightly linked to a regeneration program, both of which are evoked upon TCR stimulation and even share effector molecules, such as IFN-γ and TNF. This linkage has been speculated on recently, as a “core resident-memory T cell signature” was defined, including the upregulation of both proliferative molecules such as IL-2, but also regulatory markers such as PD-1, TIGIT, LAG-3, CD101, and IL-10, leading the authors to speculate that perhaps resident-memory T cells may play a role in limiting tissue damage (Kumar et al., 2017, 2018).

CD8 T cells can directly communicate with virtually all cells in the body via TCR/peptide-MHC-class-I interactions. This includes stromal cells and differentiated parenchymal cells, such as fibroblasts and epithelial cells. In addition to this direct communication, factors released by CD8 T cells can indirectly activate fibroblasts and epithelial cells. TNF, IFN-γ, and IL-6 are prototype factors in this respect. All have pleiotropic functions reaching from proinflammatory effects on immune cells to wound healing and proliferative effects on epithelial cells and tumor cells (Chen et al., 2007; Cheng et al., 2008; Hunter and Jones, 2015; Kuhn et al., 2014; Leppkes et al., 2014). For example, TNF has been shown to induce the expression of the EGFR-ligand TGFα in epithelial cells (Janes et al., 2006). Accordingly, we observed a CD8 T cell activation-dependent induction of the EGFR-ligands TGFα and AREG in our coculture system. We also found that human CD8 T cells in tissues such as fat and skin produce AREG under steady state. AREG production by human T cells after in vitro activation has been reported previously (Qi et al., 2012). In addition to these factors, the role of other EGFR ligands, such as TGFα and EGF, in CD8 T cell–mediated tissue remodeling requires further investigation. One could also speculate on other factors or pathways involved in tissue repair that might be produced or triggered by effector CD8 T cells, such as the release of insulin-like growth factor 1 (Richards et al., 2016; Toulon et al., 2009).

IFN-γ and TNF produced by CD8 T cells can sensitize epithelial cells to respond more vigorously with tissue regeneration to the presence of low-dose AREG, indicating that activated human CD8 T cells can prime the environment for tissue regeneration. The sensitization effect of CD8 T cells can be explained by an induced expression of the EGFR on epithelial cells as well as induced production of EGFR-ligands. Indeed, our data showed that blocking TNF during CD8 T cell activation could reduce the release of AREG and TGFα. Our data suggest that there are two EGFR-ligand-related processes acting in parallel. CD8 T cells can directly produce AREG, and effector molecules released by CD8 T cells, such as TNF, can induce the production of EGFR-ligands by epithelial cells, e.g., TGFα and AREG. Whether there is a hierarchy, or whether the exact timing of AREG production by both cell types is critical, requires further investigation. In addition, IFN-γ has been shown to have an important role in the resolution of inflammation (Feuerer et al., 2006). This is in agreement with our data showing that blocking IFN-γ, produced by human CD8 T cells, significantly reduced the tissue regenerative ability of CD8 T cells in the complex stem cell–driven organoid formation model.

While the combined cytotoxic effector and wound healing programs might be beneficial after killing virus-infected cells or tumor cells in an immune surveillance mode to limit tissue damage and restore organ function following elimination of the pathogen or tumor cell, it could pose a problem for the efficacy of immunotherapy against cancer. Based on our in vitro data, it might be beneficial to combine T cell therapy against solid tumors, such as immune checkpoint blockade or CAR T cell therapies, with targeted approaches to restrict the CD8 effector T cell–induced fibroblast or epithelial cell activation. One possibility could be the inhibition of the EGFR pathway to reduce the tissue regeneration potential in the tumor, for example, with a combination therapy of CAR T cells and Cetuximab. An additional target could be the expression of IFN-γ in CAR T cells. A recent report showed that deleting IFN-γ genetically did not compromise human CAR T cell function in hematologic malignancies (Bailey et al., 2022). Thus, removing IFN-γ from the effector program might only affect the immune suppressive and regenerative arm of CAR T cell effector responses.

Several studies showed the remarkable potential of CD4 Treg cells to promote tissue repair (Campbell and Rudensky, 2020; Panduro et al., 2016). One study investigated lung tissue, where tissue Treg cells were required to prevent severe acute lung damage following influenza infection by the production of AREG (Arpaia et al., 2015). Our data now show that human CD8 T cells likewise have an antigen-specific tissue regenerative ability, a feature that is even extended to the tumor cell -TIL, and tumor cell - CEA–CAR interaction. While Treg cells recognize self-peptides and respond by suppressing further inflammation and releasing tissue repair–promoting factors (Delacher et al., 2021), CD8 T cells might use their antigen-specific cytotoxic effector machinery to destroy target cells and, as a linked program, start to induce the resolution and restoration of tissue damage as an early player, mediated presumably by reprogramming the damage-surrounding cells (fibroblast, epithelial cells, and tissue stem cells) for tissue regeneration. Factors released in this early phase, such as AREG, TNF, and IFN-γ, might also activate other cells with tissue regeneration potential, such as tissue-resident Treg cells or macrophages relevant for the later phase of tissue reconstitution. However, in the case of chronic CD8 T cell activation, this regenerative cascade can potentially also cause fibrosis, as has been reported in the thyroid and lung (Brodeur et al., 2015; Yu et al., 2011). The conceptual differences between Treg cells, which are a stable lineage of anti-inflammatory and immunosuppressive cells, and CD8 T cells, usually proinflammatory and cytotoxic, now require a careful dissection of regeneration and remodeling programs versus cytotoxic and inflammatory programs.

## Materials and methods

### Ethics statement

Human skin and subcutaneous adipose tissue used for scRNA/scTCR-seq, RNA-seq, and flow cytometry were obtained from healthy female donors undergoing abdominoplasty procedures after weight loss or epigastric hernia repair after multiple pregnancies. Human primary liver tumors (two cholangiocellular carcinomas and one hepatocellular carcinoma) as well as surrounding healthy liver tissues and PBMCs used for cytokine expression analysis were obtained from three patients (two female and one male) undergoing a major liver resection. Collection of skin, fat, and blood samples from donors was performed after ethical approval by the local ethical committee (Regensburg University, reference number 19-1453-101) and signed informed consent. Collection of primary liver tumors, surrounding liver tissue, and blood samples from tumor patients was performed after ethical approval of the local ethical committee (Regensburg University, reference number 18-1075-101) and signed informed consent. Human gallbladder tissue (2 cm^2^), used for the generation of organoids, was obtained from donors during routine cholecystectomy after ethical approval of the local ethical committee (Regensburg University, reference number 16-1015-101) and signed informed consent. PBMCs for CD8 T cell enrichment were isolated from leukocyte reduction chambers from healthy donors donating thrombocytes. Collection of immune cells from those donors was performed in compliance with the Helsinki Declaration after ethical approval by the local ethical committee (Regensburg University, reference number 13-0240-101 and 19-1414-101) and signed informed consent.

### Combined in vitro cytotoxicity and generation of SNs for wound healing assays or 3D tumor spheroid growth assays with ex vivo isolated TILs

Human TIL209 cells (Eisenberg et al., 2013; Jin et al., 2012; Khandelwal et al., 2015; Machlenkin et al., 2008) were thawed and rested o/n in complete lymphocyte (CLM) medium (RPMI supplemented with 10% AB Human Serum, 0.01% 50 mM 2-MercaptoEtOH, 1% 1 M HEPES, and 1% Penicillin/Streptomycin) supplemented with 3,000 U/ml IL-2, 5 ng/ml IL-7, and 5 ng/ml IL-15. Subsequently, 100,000 TILs/well were seeded on a layer of confluent M579 cells in TexMACS medium supplemented with 1% Penicillin/Streptomycin. As a negative control, M579 cells without TILs were cultured in TexMACS with 1% Penicillin/Streptomycin. Cytotoxicity was measured by adding Incucyte Caspase 3/7 dye for apoptosis (Essen Bioscience) to the wells at a dilution of 1:6,000 and placing the plate in the Incucyte SX5 Live-Cell Analysis Instrument (Essen Bioscience), and scans of the wells were scheduled for every 60 min for 18 h. The amount of green signal detected was calculated with Incucyte Scratch Wound Cell Migration and Invasion System software (Essen Bioscience) and statistical significance was established with GraphPad Prism (GraphPad software). SNs were harvested and used for ELISA, 3D tumor spheroid growth assay, and in vitro wound healing assays.

### Generation of influenza-specific T cells

Human peripheral blood from HLA-A2–specific donors (Volpin et al., 2020) was separated by Ficoll gradient centrifugation and pre-enriched with anti-human CD8 beads and expanded in the presence of the influenza peptide (GILGFVFTL; ProImmune) for 14 d. The CD8^−^ fraction was irradiated and used as feeder cells for 1 wk until being replaced by irradiated T2 cells. After 1 and 8 d, 100 U/ml IL-2 and 5 ng/ml IL-15 were added. Cells were sorted for influenza specificity by pentamer staining (GILGFVFTL-APC, #P007-0A-E; ProImmune) on day 14 before expanding the cells in medium containing 3,000 U/ml IL-2 and 30 ng/ml anti-CD3 for 14 d. Influenza-specific T cells were frozen until use in subsequent assays.

### Combined in vitro cytotoxicity/proliferation assays and generation of SNs for ELISA, 3D tumor spheroid growth assay, and wound healing with influenza-specific T cells

Human influenza-specific T cells were thawed and rested o/n in CLM medium. 30,000 MRC-5 cells/well or 9,000 MRC-5 cells and 21,000 HaCaT cells/well were seeded and grown to full confluency o/n before pulsing the cells with varying concentrations of influenza peptide (0, 1, 10, or 100 ng/ml) for 1 h. Cells were washed twice before adding 30,000 influenza-specific T cells in TexMACS with 2% Penicillin/Streptomycin. For TNF inhibition, 10 µg/ml anti-human TNF (Invivogen) or hIgG (Jackson Immuno Research) was added to the coculture simultaneously to adding the influenza-specific T cells. For IFN-γ inhibition, 10 µg/ml anti-human IFN-γ (Biozol) or mIgG (Jackson Immuno Research) was added to the coculture simultaneously to adding the influenza-specific T cells. For cytotoxicity/HaCaT proliferation assays, HaCaT cells were stained with Vybrant DiD Cell-Labeling Solution (Thermo Fisher Scientific) according to the manufacturer’s protocols prior to seeding. Cytotoxicity and HaCaT proliferation were measured as described above. For preactivation experiments, cells were washed 3× with TexMACS medium after 20 h cocultivation to remove all T cells. Bystander cells were then allowed to produce SN for 24 h before harvesting of SNs for subsequent assays. SNs were harvested and used for ELISA, 3D tumor spheroid growth assays, and in vitro wound healing assays.

### Tissue digestion for flow cytometry and FACS sorting of human T cells and fibroblasts

To isolate T cells and fibroblasts from human skin and subcutaneous fat tissue, skin and underlying fat were first mechanically separated, followed by tissue-individual preparation steps. Fat was cut into small pieces and digested for 90 min at 37°C (base medium DMEM [#41965; Gibco], 1 mg/ml collagenase type II [#C6885; Sigma-Aldrich], 20 µg/ml DNAse I [#11284932001; Roche], 20 mg/ml bovine serum albumin [#A4503; Sigma-Aldrich], 10 mM HEPES) on a MACSmix tube rotator, followed by filtration and centrifugation steps as well as red blood cell lysis using ACK lysis buffer (#A1049201; Gibco).

To isolate T cells from human skin tissue, skin was cut into small pieces and digested (base medium DMEM [#41965; Gibco], 4 mg/ml collagenase type IV [#C5138; Sigma-Aldrich], 10 µg/ml DNAse I [#11284932001; Roche], 2% fetal bovine serum, and 10 mM HEPES). Digestion was performed directly in a GentleMACS C tube ((#130-093-237; Miltenyi Biotec)) and the program “37_C_Multi_H” for 90 min on a gentleMACS Dissociator, followed by centrifugation and filtration steps. Dead cell removal was performed with a dead cell removal kit. More detailed protocols about T cell isolation from human skin and fat tissues are available (Delacher et al., 2021).

To isolate T cells from human liver and liver tumor tissue, samples were cut into small pieces and digested (base medium DMEM [#41965; Gibco], 1 mg/ml collagenase type IV [#C5138; Sigma-Aldrich], 25 µg/ml DNAse I [#11284932001; Roche], 10% fetal bovine serum, and 10 mM HEPES). Digestion was performed directly in a GentleMACS C tube. Tissues were minced using the program “h.tumor” on the GentleMACS and were subsequently attached to a MACSmix tube rotator and placed at 37°C for 60 min. Digestion was stopped and cells were washed with PBS + 2 mM EDTA. A single-cell solution was obtained by centrifugation and filtration steps as well as red blood cell lysis using ACK lysis buffer (# A1049201; Gibco).

### PBMC isolation and pre-enrichment of blood lymphocytes

To isolate T cells from human blood, leukocyte reduction chambers (provided by Transfusion Medicine, University Clinics Regensburg) were used. Leukocytes were first diluted three times with Dulbecco’s Balanced Salt Solution (DPBS) (#14190-094; Gibco) and the resulting blood and DPBS mixture was split into two fractions and underlayed with an equal amount of Pancoll (#P04-601000; PAN biotech). Samples were centrifuged at 1,000 × *g* for 20 min at room temperature (RT), with acceleration set to 4 and brake to 0. The PBMC layer was isolated and washed twice by centrifugation steps. Cells were pre-enriched with biotinylated anti-human CD8 (clone HIT8a; Biolegend), followed by column-based magnetic separation with anti-biotin ultrapure microbeads (#130-105-637; PAN biotech) following the manufacturer’s protocol.

### Preparation of samples for FACS sorting or flow cytometry

T cells or fibroblasts were isolated and pre-enriched as described in the previous sections. Cells were stained in 1.5 ml Eppendorf tubes or 96-well plates in FACS buffer (2% FCS in PBS). Surface staining was performed at 4°C for 20 min in 50–100 μl staining volume. Antibodies were used, if not indicated otherwise, as recommended by the manufacturer. The following anti-human antibodies were used for surface staining at a dilution of 1:200: CD3 (OKT3/SK7), CD4 (OKT4/L200/SK3/A161A1), TCR-β chain (IP26), CD8 (RPA-T8/HIT8a), CD19 (HIB19), CD140a (16A1), CD45RA (HI100), CD45RO (UCHL1), CD45 (HI30/2D1/REA747), CD14 (MφP9/HCD14), CD39 (TU66), CD279 (REA1165), CD206 (19.2), TIGIT (MBSA43/A15153G), HLA-A2 (BB7.2), CD90 (5E10), PDPN (NC-08), EPCAM (9C4), and CD31 (WM59).

For intracellular staining of cytokines, tissue or PBMC single-cell suspensions were restimulated with cell stimulation cocktail including transport inhibitor (TI) (00-4975-93; eBioscience) or just TI (00-4980-93; eBioscience) for 4 or 16 h at 37°C. After stimulation, cells were washed, and surface staining was performed as mentioned above. Intracellular staining of stimulated cells was performed with BD Cytofix (#554655; BD) in combination with BD Phosflow perm buffer (#557885; BD) according to the manufacturer’s protocol with the following adaptations: intracellular staining steps were performed for 60 min at RT. The following antibodies were used for intracellular staining of human cytokines: TNF (Mab11) at a dilution of 1:50, IFN-γ (B27/4S.B3) at a dilution of 1:50, and AREG (AREG559) at a dilution of 1:50.

Intracellular staining for transcription factors was performed with the Foxp3/Transcription Factor Buffer Set (00-5521-00; eBioscience) according to the manufacturer’s protocol with the following adaptations: intracellular staining steps were performed for 60 min at RT. The following antibodies were used for intracellular staining: TOX (TXRX10) at a dilution of 1:20 and BATF (D7C4) at a dilution of 1:200. For BATF staining, secondary intracellular staining was performed with anti-rabbit AF647 (Cat#4414; Cell Signaling) or anti-rabbit AF488 (Cat#4412; Cell Signaling) antibody at 1:400. Dead cells were excluded with a fixable live/dead dye (Fixable Viability Dye eFlour780, Cat# 65-0865-14; eBioscience).

### Combined in vitro cytotoxicity assay and generation of SNs for ELISA and wound healing assay with CEA-CAR T cells

Human peripheral blood was separated by Ficoll gradient centrifugation, and CD8 T cells were enriched for by positive magnetic column selection with anti-human CD8 beads. Cells were rested at a concentration of 80,000 cells/well in TexMACS medium supplemented with 1% Penicillin/Streptomycin, 100 U/ml IL-2, and TransAct o/n. For virus production, AMPHO-Phoenix-293T HEK cells were transfected with either a murine stem cell virus (MSCV) vector containing the construct for the CEA-CAR or the empty MSCV vector. Efficient transfection was confirmed by FACS measurement of CD90.1 expression in HEK cells. After 48 h, virus-containing SNs were harvested and CD8 T cells were infected in the presence of polybrene for 6 h. After infection, CD8 T cells were expanded for 72 h in TexMACS medium containing 100 U/ml IL-2 and TransAct. Subsequently, cells were rested for 24 h in TexMACS Medium with 50 U/ml IL-2 and 1% Penicillin/Streptomycin before sorting for viable CD8^+^CD90.1^+^ T cells. CEA-CAR T cells, T cells infected with the negative vector, or no T cells were seeded on a confluent layer of A549 cells with a stable expression of CEA. Cytotoxicity was measured as described above. SNs were harvested and used for ELISA and in vitro wound healing assays.

### In vitro wound healing assay using different SNs or cytokines and HaCaT cells

The human keratinocyte cell line HaCaT (RRID: CVCL_0038) was grown in RPMI supplemented with 10% FCS and 1% Penicillin/Streptomycin. 20,000 cells/well were seeded in an ImageLock plate (Cat#4379; Essen Bioscience) and were allowed to settle and expand for 18 h at 37°C. Wounds were made with a WoundMaker (Essen Bioscience) and cells were washed 3× with TexMACS medium supplemented with 1% Penicillin/Streptomycin to remove all traces of FCS. SNs were diluted at a ratio of 1:4 and added to HaCaT cells, which were then placed at 37°C. For inhibition experiments, Cetuximab (Selleckchem) or hIgG (Jackson Immuno Research) were added to the SNs at a final concentration of 10 µg/ml and incubated at RT for 15 min prior to adding the SNs to the HaCaT cells. For cytokine experiments, either (i) 100 ng/ml AREG, (ii) 5 ng/ml AREG, (iii) 5 ng/ml TNF and 1 ng/ml IFN-γ, or (iv) 5 ng/ml AREG, 5 ng/ml TNF, and 1 ng/ml IFN-γ were added instead of SNs. Culture plates were placed in the Incucyte SX5 Live-Cell Analysis Instrument (Essen Bioscience) and scans of the wells were scheduled for every 60 min for 48 h. Relative wound density was calculated with Incucyte Scratch Wound Cell Migration and Invasion System software (Essen Bioscience). Relative wound density values were analyzed and statistical significance was established with GraphPad Prism (GraphPad software).

### In vitro 3D tumor spheroid growth assay using different SNs or cytokines and HCT116 cells

The human colon cancer cell line HCT116 (RRID:CVCL_0291) was seeded at a concentration of 4,000 cells/well in Costar Ultra-Low Attachment Multiple Well Plates (CLS7007-24EA; Sigma Aldrich) in TexMACS medium supplemented with 1% Penicillin/Streptomycin. 1 d after seeding, initial spheroid formation occurred and spheroids were stimulated by replacing half of the medium with either SN generated as described above or different cytokines (IL-10, IFN-γ, TNF all purchased from Peprotech or equal amounts of 0.1% BSA in PBS as a carrier) at a final concentration of 10 ng/ml. 5 d later, another half-medium change with SN or cytokines was performed. During culture, plates were placed in the Incucyte SX5 Live-Cell Analysis Instrument (Essen Bioscience) and scans of the wells were scheduled for every 60 min. The size of the largest brightfield object was calculated with Incucyte Scratch Wound Cell Migration and Invasion System software (Essen Bioscience) and statistical significance was established with GraphPad Prism (GraphPad software).

### Stimulation of human fibroblasts with SN of tissue CD8 T cells

CD8 T cells were isolated from human skin tissue as described above and cultured at a concentration of 150,000 cells/well with anti-CD3/CD28 beads at a ratio of 1:2 and 100 U/ml IL-2 in TexMACS medium at 37°C, 5% CO_2_. After 24 h, medium was collected and stored at −80°C until stimulation. As a negative control, medium with additives but without cells was collected. SNs were stored at −80°C until use.

Fibroblasts were isolated from human skin tissue as described above, and viable, CD45^+^, CD31^−^, EPCAM^−^, PDPN^+^, CD90^+^, and CD140a^+^ cells were FACS sorted. Isolated cells were subsequently cultured in flat-bottom 96-well plates at 50,000 cells/well in fibroblast cultivation medium (RPMI supplemented with 5% FCS, 2 mM *L*-glutamine, 10 mM HEPES, pH 7.4, 1 mM sodium pyruvate, 1% Penicillin/Streptomycin, and 0.05 mM 2-MercaptoEtOH) at 37°C, 5%CO_2_ for 14 d with intermittent medium changes. After initial culture, fibroblasts were washed 3× with PBS and then stimulated o/n with autologous CD8 cell SN or empty SN diluted 1:4 in TexMACS medium. Fibroblasts were then harvested in RNA lysis buffer for subsequent bulk RNA-seq.

### Generation of human bile duct organoids (ECO)

For organoid generation, gallbladder tissue (2 cm^2^ or less) was washed two times with cold Earle’s Balanced Salt Solution (EBSS; Gibco), cut into small pieces, and digested in 4 ml digestion solution: 25 mg/ml collagenase from *Clostridium histolyticum* (Sigma-Aldrich) in EBSS for 20 min at 37°C with soft shaking and filtered through a 70-μM Nylon CellStrainer. Dissociated cells were centrifuged at 1,500 rpm for 5 min at 4°C and washed twice with Base-medium (100 U/ml penicillin, 100 μg/ml streptomycin, 1 μg/ml AmphotericinB, 2 mM *L*-Glutamine, and 50 mM HEPES). Organoid cultures were established as previously published (Huch et al., 2015). In brief, cell pellets were resuspended in organoid culture medium mixed with matrigel (Corning) in a 50:50 ratio. Matrigel was allowed to solidify for 15 min at 37°C before adding organoid culture medium. Organoid culture medium was based on ADV/DMEM-F12 (Gibco) supplemented with 1 M HEPES (Sigma-Aldrich), 100 mM *L*-glutamine (Sigma-Aldrich), 3.6% Anti-Anti (Gibco), 1% N2 serum-free supplement (Gibco), 1% B27 serum-free supplement (Gibco), 1 mM N-acetyl L-cysteine (Sigma-Aldrich), 10 nM gastrin I (Sigma-Aldrich), and the following growth factors: 1 µg/ml of recombinant human R-spondin 1 (Peprotech), 10 mM nicotinamin (Sigma-Aldrich), 5 μM A83.01 (Peprotech/BioGems), 10 μM Forskolin (R&D), 50 ng/ml human EGF (Peprotech), 50 ng/ml human HFG (Peprotech), and 100 ng/ml human FGF-10 (Peprotech). For the first 72 h after thawing, 10 μM of Y-27632 (Peprotech) was added to the media and only 25 ng/ml HGF were used. Media was changed every 3–4 d. Organoids were split every week by mechanical dissociation into small fragments and transferred to fresh matrigel.

### Coculture of organoids and CD8 T cells or CD8 T cell–derived SN

Human peripheral blood was separated by Ficoll gradient centrifugation and pre-enriched with anti-human CD8 beads as described above. Cells were purified using magnetic columns followed by FACS of viable CD8^+^ T cells. 100,000 cells/well were seeded in TexMACS medium supplemented with 1% Penicillin/Streptomycin and activated with Transact (1:100). After 4 d, cells were harvested and rested in TexMACS medium with 100 U/ml IL-2 for 3 d. Subsequently, 200,000 T cells were stimulated with Transact o/n before harvesting the cells or cell-derived SN. SN of stimulated CD8 T cells was harvested and cells were washed 3× with organoid culture medium without growth factors. Organoids were harvested and washed with PBS to remove matrigel. For coculture, organoids and CD8 T cells were mixed in a ratio of 20 organoids/10,000 CD8 T cells in organoid culture medium without growth factors. The mixes were pelleted and resuspended in a 50:50 mixture of matrigel and organoid culture medium supplemented with growth factors as described above, but without EGF. Then, 25 μl of the mixture was seeded in the center of the wells of a 48-well plate (Corning) and incubated at 37°C for 15 min to allow matrix solidification and dome formation. Finally, 300 μl of organoid culture supplemented with growth factors and 100 U/ml IL-2 was added and cocultures were incubated at 37°C and 5% CO_2_ in the Incucyte SX5 Live-Cell Analysis Instrument. Scans of the wells were scheduled for every 8 h for 5 d. For experiments using CD8 T cell–derived SN, the SN was diluted 1:8 in organoid culture medium supplemented with growth factors and added at 300 μl per well. As a negative control for cell-derived SN, TexMACS medium with Transact was used. For inhibition experiments, 10 µg/ml anti-human TNF (Invivogen) or 10 µg/ml anti-human IFN-γ (Biozol) or the respective controls, hIgG (Jackson Immuno Research) and mIgG (Jackson Immuno Research), was added at the start of the coculture, both into the matrigel as well as in the culture medium.

### Analysis of organoid images

Bright-field images of organoids were acquired every 8 h over a period of 120 h by the Incucyte SX5 Live-Cell Analysis Instrument (Essen Bioscience). Processing and merging of z-stacks was performed by the organoid module of Incucyte system according to the manufacturer’s instructions (Sartorius AG). The TissueFAXSiPlus system (TissueGnostics) was used for endpoint analysis. Z-stacks from four focal planes were merged to generate bright-field images of organoids. Bright-field image analysis and subsequent quantification of organoid size and number was performed by StrataQuest (version 7.1.1.129; TissueGnostics; Stüve et al., 2023).

### ELISA

ELISAs were purchased from R&D Systems and performed according to the manufacturer’s specifications.

### Flow cytometry and FACS sorting of T cells from blood and tissues

T cells were isolated, pre-enriched, and stained as described previously. Afterward, samples were filtered with a 40-µM filter unit and acquired on a BD FACSymphony, a BD FACSCelesta, or a BD FACSFusion flow cytometer. BD CS&T beads were used to validate machine functionality. Fluorescence spillover compensation was performed with lymphocytes stained with CD4 (OKT4) in the respective colors. Flow cytometry data were analyzed using BD FlowJo (Version 10.6.2). Sorting was performed with a BD FACSAriaII or BD FACSFusion cell sorter with an 85- or 70-µm nozzle. Post-sort quality controls were performed as applicable. For scRNA-seq, target cells were sorted into 500 μl 10%FCS-PBS. For bulk RNA-seq, cells were sorted directly into 500 μl RLT+ lysis buffer (RNEasy Plus Micro Kit #74034; Qiagen). For cultivation experiments, cells were sorted directly into cell culture medium. All procedures were performed in DNA low-bind tubes (#0030108051; Eppendorf) or 15-ml tubes. For cytokine restimulation experiments, cells were incubated with either 1X cell stimulation cocktail with transport inhibitors or transport inhibitor only (both eBiosciences) in cell culture medium for 4 h at 37°C. Afterward, cells were surface stained, followed by intracellular staining with the Foxp3 Fix/perm kit.

### Generation of SN for in vitro wound healing assay from peripheral blood PD1^+^TIGIT^+^ CD8 T cells

Human peripheral blood was separated by Ficoll gradient centrifugation and pre-enriched with anti-human CD8 beads as described above. Cells were purified using magnetic columns followed by fluorescence-activated cell sorting of viable, CD8^+^CD45RA^−^CD45RO^+^PD1^+^TIGIT^+^ T cells. 100,000 cells/well were seeded on a layer of ∼30% confluent MRC-5 cells in TexMACS medium supplemented with 1% Penicillin/Streptomycin and activated with anti-CD3/CD28 beads (4:1) o/n before harvesting the SN. As a negative control, SN of MRC-5 cells cultured o/n in TexMACS medium and stimulated anti-CD3/CD28 beads were used.

### Generation of SN for in vitro wound healing assay from peripheral blood CD8 T cell subpopulations

Human peripheral blood was separated by Ficoll gradient centrifugation and pre-enriched with anti-human CD8 beads as described above. Cells were purified using magnetic columns followed by fluorescence-activated cell sorting of viable, CD8^+^CCR7^+^CD45RA^+^ (Tnaive), CD8^+^CCR7^+^CD45RA^−^ (Tcm), CD8^+^CCR7^−^PD1^+^TIGIT^+^ (Tem) cells. 100,000 cells/well were seeded in TexMACS medium supplemented with 1% Penicillin/Streptomycin and activated with anti-CD3/CD28 beads (4:1) o/n before harvesting the SN. As a negative control, SN of MRC-5 cells cultured o/n in TexMACS medium and stimulated anti-CD3/CD28 beads were used.

### CRISPR-Cas9 KO of AREG in human CD8 T cells for EdU assay

Human peripheral blood was separated by Ficoll gradient centrifugation and pre-enriched with anti-human CD8 beads as described above. Cells were purified using magnetic columns followed by fluorescence-activated cell sorting of viable CD8^+^ T cells. Cells were cultured with Transact and 100 U/ml IL-2 in TexMACS medium at 37°C, 5% CO_2_ for 2 d prior to transfection.

Guide RNA was generated by mixing tracrRNA (Cat. No. 1072534; Integrated DNA Technologies) and AREG crRNAs (sequences: 5′-TCT​AGT​AGT​GAA​CCG​TCC​T-3′, 5′-GAC​CTC​AAT​GAC​ACC​TAC​TC-3′, and 5′-GAT​AAC​GAA​CCA​CAA​ATA​CC-3′) or tracrRNA and scrambled crRNAs (Cat. No. 1072544; Integrated DNA Technologies) in equimolar concentrations, heated to 95°C for 5 min and allowed to cool down to RT. Guide RNA mixes are combined with Cas9 Enzyme (Cat. No. 1081060; Integrated DNA Technologies) and incubated at RT for 10–20 min to form the RNP complex. Transfection was performed using the NEON transfection instrument (settings: 1,600 V, 10 ms pulse width, 3 pulses; Thermo Fisher Scientific) in the presence of an electroporation enhancer (Cat. No. 1075915; Thermo Fisher Scientific) using the Neon Transfectionsystem 10 μl Kit (Cat. No. MPK1096; Thermo Fisher Scientific). Cells were cultured with 100 U/ml IL-2 and Transact for 2 d before resting in medium with 50 U/ml IL-2. To determine efficacy of CRISPR KOs, cells were stimulated with PMA/ionomycin in the presence of transport inhibitors and stained for flow cytometry as described above. For the EdU assay, cells were stimulated with 100 U/ml IL-2 and transact 1:100 o/n after 2 d of resting. EdU assay was carried out using the Click-iT EdU Alexa Fluor 488 Flow Cytometry-Assay-Kit (Cat. No. C-10425; Thermo Fisher Scientific) according to the manufacturer’s instructions. Briefly, cells were incubated with 10 µM EdU dissolved in PBS for 1 h before harvesting cells with Trypsin/EDTA and staining with viability dye. Cells were then fixed with fixative component for 15 min at RT in the dark, followed by washing and permeabilization steps. The Click-it reaction was then performed using fluorescent dye azide AF488 for 30 min. Cells were then washed in permeabilization buffer and stained with DAPI (1 µg/ml, Cat. No. 10236276001; Sigma-Aldrich) for 2 min at RT in the dark. Cells were washed, filtered, and acquired on the BD FACSCelesta.

### scRNA/TCR-seq of human blood and tissue T cells

Human T cells were isolated, pre-enriched, stained, and sorted as described previously. Sort gates are shown in Fig. S2 A. From fat tissue of donor RT1, we sorted 40,000 CD45^+^Dead^−^CD14^−^CD19^−^CD3^+^TCRβ^+^CD4^−^CD8^+^ T cells. From skin tissue of donor 6, we sorted 40,000 CD45^+^Dead^−^CD14^−^CD19^−^CD3^+^TCRβ^+^CD4^−^CD8^+^ T cells. From peripheral blood of donor 6, we sorted 50,000 CD45^+^Dead^−^CD14^−^CD19^−^CD3^+^TCRβ^+^CD4^−^CD8^+^ T cells. From fat tissue of donor RT2, we sorted 32,000 CD45^+^Dead^−^CD14^−^CD19^−^CD3^+^TCRβ^+^CD4^−^CD8^+^ T cells. From skin tissue of donor 7, we sorted 40,000 CD45^+^Dead^−^CD14^−^CD19^−^CD3^+^TCRβ^+^CD4^−^CD8^+^ T cells. From peripheral blood of tissue donor 7, we sorted 50,000 CD45^+^Dead^−^CD14^−^CD19^−^CD3^+^TCRβ^+^CD4^−^CD8^+^ T cells. After sorting, cells were centrifuged (1,000 × *g*, 5 min, 4°C) and reconstituted in 38 μl 10% FCS-PBS buffer. Master Mix (Chromium Next GEM Single Cell 5′ Gel bead V1.1, #1000165; 10X Genomics) was added to the cells and samples were loaded on 10X Chromium Next GEM Chip G (#1000120; 10X Genomics). GEM incubation was performed for 45 min at 53°C according to the manufacturer’s protocol. Single-cell libraries were prepared as per the manufacturer’s protocol (Chromium Next GEM Single Cell 5′ Library and Gel Bead Kit v1.1). scRNA libraries were sequenced on an Illumina NextSeq 550 with NextSeq 500/550 High Output Kit v2.5 (75 cycles). scTCR libraries were sequenced on an Illumina NextSeq 550 with NextSeq 500/550 Mid Output Kit v2.5 (300 cycles, Cat# 20024905; Illumina).

### Analysis of scTCR-seq data

Fastq files were processed using Cell Ranger (version 3.1.0) based on 10X Genomics provided VDJ reference (version 3.1.0). Clones from different samples were matched by TCR α and β nucleotide sequences. Clonal abundance pie charts were generated using ggplot2 (version 3.3.4) and R (version 4.0.4). Different TCR numbers between samples and respective pie charts are based on the removal of certain clusters.

### Analysis of scRNA-seq data

Fastq files were processed using Cell Ranger (version 3.1.0) based on 10X Genomics–provided hg19 reference genome (version 3.0.0). Cell Ranger was run per sample (using cellranger count). Downstream analysis was performed per donor, and the R package Seurat (Butler et al., 2018; version 4.0.3) together with R (version 4.0.0) was used. Cells with fewer than 500 transcript features were discarded as well as cells exceeding a 5% threshold of mitochondrial transcripts. The data was log normalized (using NormalizeData) and scaled (using ScaleData). Highly variable genes were identified (using FindVariableFeatures) with default parameter settings and principle components calculated (using RunPCA[npcs = 40]). UMAP dimensionality reduction was performed (using RunUMAP). Differential gene expression analysis was performed (using FindMarkers[logfc.threshold = 0, ... ]).

### Mapping of RNA-seq data, statistical evaluation, and plotting

For all samples, low-quality bases were removed with Fastq_quality_filter from the FASTX Toolkit 0.0.13 with 90% of the reads needing a quality phred score > 20. Homertools 4.7 (Heinz et al., 2010) were used for PolyA-tail trimming and reads with a length < 17 were removed. PicardTools 1.78 were used to compute the quality metrics with CollectRNASeqMetrics. With STAR 2.3 (Dobin et al., 2013), the filtered reads were mapped against human genome 38 using default parameters. Count data and reads per kilo base per million mapped reads (RPKM) tables were generated by mapping filtered reads against union transcripts using a custom pipeline. Mapping was carried out with bowtie2 version 2.2.4 (Langmead and Salzberg, 2012) against union human genes: every gene is represented by a union of all its transcripts (exons). The count values (RPKM and raw counts) were calculated by running CoverageBed from Bedtools v2.26.0 (Quinlan and Hall, 2010) of the mapped reads together with the human annotation file (Ensembl 90) in a gtf format and parsing the output with custom perl scripts. The input tables containing the replicates for groups to compare were created by a custom perl script. For the pairwise comparisons DESeq2 (Love et al., 2014), DESeqDataSetFromMatrix was applied, followed by estimateSizeFactors, estimateDispersions, and nbinomWald testing. MA plots were generated using the plotMA function of DESeq2 using all data. The principle component analysis (PCA) plots were generated by DESeq2’s plotPCA after transforming the counts using varianceStabilizingTransformation and selecting the genes from the DESeq2 result according to the adjusted P value (Padj < 0.001). RPKM table and statistical results are provided in supplementary tables.

### GSEA

For GSEA, we first obtained the human hallmark gene set collection from MSigDB (Subramanian et al., 2005; Liberzon et al., 2011). We then sorted genes from the Deseq2 results by their log_2_FC and used the function “GSEA(eps = 1e-50, pvalueCutoff = 0.05, PAdjustMethod = “BH”)” from the clusterProfiler R package (v.3.16.1) to calculate gene set enrichments. Finally, the top 15 enriched terms were plotted using “dotplot(showCategory = 15).”

### Reference-based cell annotation

SingleR (Aran et al., 2019; version 1.0.6) was used for reference-based cell annotation together with the Monaco immune reference atlas from the “celldex” package (version 1.0.0). For annotation of CD8 T cells from healthy human tissues, the reference was reduced to CD8 T cells, whereas the complete T cell subset was used for annotation of the human HCC T cell dataset. Normalized gene activities were used as input to SingleR, and cells were assigned according to SingleR’s “pruned.labels” output.

### Quantification and statistical analysis

Data were analyzed with Prism software or algorithm. Statistical details are indicated in the figure legend. Population size is described in the figure legend.

### Online supplemental material

Fig. S1 shows wound healing scratch assay. Fig. S2 shows scRNA/TCR-seq of human CD8 T cells with donor RT1. Fig. S3 shows cytokine expression and wound healing potential of blood-derived CD8 T cells. Fig. S4 shows that human CD8 T cells can promote tumor growth in vitro. Fig. S5 shows that human CD8 T cells can promote tissue regeneration in organoids. Table S1 shows bulk RNA-seq results. This table contains data from RNA-seq of fibroblast (MRC-5) cells stimulated o/n with SN of MRC-5 cells pulsed with varying concentrations of influenza peptide and cultured with influenza-specific T cells o/n (*n* = 3). RPKM table, components of PCA, and Deseq2 comparisons are listed. Table S2 shows bulk RNA-seq results. This table contains data from RNA-seq of primary human fat fibroblasts stimulated with SN generated from autologous CD8 T cells isolated from fat tissue and stimulated with IL-2 and beads o/n or empty medium ctrl (*n* = 4). RPKM table and Deseq2 comparisons are listed. Table S3 shows bulk RNA-seq results. This table contains data from RNA-seq of epithelial cells (HaCaT) stimulated o/n with SN of MRC-5 cells pulsed with varying concentrations of influenza peptide and cultured with influenza-specific T cells o/n (*n* = 4). RPKM table, components of PCA, and Deseq2 comparisons are listed. Table S4 shows scRNA/TCR-seq results. This table contains scRNA/TCR-seq results and analyses used in this paper (Table 1 shows donor RT1 blood CD8^+^ TCR clonotype information with clonotype ID, sample name, internal identifier, frequency of TCR clone, proportion, CDR3 sequence [amino acid], CDR3 sequence [nucleotide], and barcode; Table 2 shows donor RT1 skin CD8^+^ TCR clonotype information; Table 3 shows donor RT1 fat CD8^+^ TCR clonotype information; Table 4 shows donor RT2 blood CD8^+^ TCR clonotype information; Table 5 shows donor RT2 skin CD8^+^ TCR clonotype information; Table 6 shows donor RT2 fat CD8^+^ TCR clonotype information). Video 1 shows organoid growth from Fig. 7 A in the control group “ECO only” over time. Video 2 shows organoid growth from Fig. 7 A in the coculture of “ECOs and 10k CD8 T cells” over time. Video 3 shows organoid growth from Fig. 7 B in the control group “ECO only” over time. Video 4 shows organoid growth from Fig. 7 B in the culture of “ECO with CD8 SN” over time.

## Supplementary Material

Table S1 shows bulk RNA-seq results of fibroblast (MRC-5) cells stimulated o/n with SN of MRC-5 cells pulsed with varying concentrations of influenza peptide and cultured with influenza-specific T cells o/n.

Table S2 shows bulk RNA-seq results of primary human fat fibroblasts stimulated with SN generated from autologous CD8 T cells isolated from fat tissue and stimulated with IL-2 and beads o/n or empty medium ctrl.

Table S3 shows bulk RNA-seq results of epithelial cells (HaCaT) stimulated o/n with SN of MRC-5 cells pulsed with varying concentrations of influenza peptide and cultured with influenza-specific T cells o/n.

Table S4 shows scRNA/TCR-seq results and analyses used in this paper.

## Data Availability

The data in the figures are available in the published article and in the online supplemental material. The accession numbers for human scRNA-seq and scTCR-seq (Figs. 3 and S2) data reported in this paper are European Genome-phenome Archive EGAD00001010007 and EGAD00001010005, respectively. The accession number for human primary fibroblast bulk RNA-seq data (Fig. 2 B) is EGAD00001010004. The accession number for MRC-5 and HACAT bulk RNA-seq data (Fig. 2, A and C) reported in this paper is Gene Expression Omnibus GSE223989.
